# Hits Discovery on the Androgen Receptor: In Silico Approaches to Identify Agonist Compounds

**DOI:** 10.3390/cells8111431

**Published:** 2019-11-13

**Authors:** Manon Réau, Nathalie Lagarde, Jean-François Zagury, Matthieu Montes

**Affiliations:** Laboratoire GBCM, EA 7528, Conservatoire National des Arts et Métiers, HESAM Université, CEDEX, F-75003 Paris, France; manon.reau@lecnam.net (M.R.); nathalie.lagarde@lecnam.net (N.L.); zagury@cnam.fr (J.-F.Z.)

**Keywords:** androgen receptor, agonist compounds, virtual screening, docking, pharmacophore modeling, therapeutic research, public health

## Abstract

The androgen receptor (AR) is a transcription factor that plays a key role in sexual phenotype and neuromuscular development. AR can be modulated by exogenous compounds such as pharmaceuticals or chemicals present in the environment, and particularly by AR agonist compounds that mimic the action of endogenous agonist ligands and whether restore or alter the AR endocrine system functions. The activation of AR must be correctly balanced and identifying potent AR agonist compounds is of high interest to both propose treatments for certain diseases, or to predict the risk related to agonist chemicals exposure. The development of *in silico* approaches and the publication of structural, affinity and activity data provide a good framework to develop rational AR hits prediction models. Herein, we present a docking and a pharmacophore modeling strategy to help identifying AR agonist compounds. All models were trained on the NR-DBIND that provides high quality binding data on AR and tested on AR-agonist activity assays from the Tox21 initiative. Both methods display high performance on the NR-DBIND set and could serve as starting point for biologists and toxicologists. Yet, the pharmacophore models still need data feeding to be used as large scope undesired effect prediction models.

## 1. Introduction

The androgen receptor (AR) is a transcription factor involved in various physiological processes including male sexual phenotype, the development and maintenance of musculoskeletal and cardiovascular systems, as well as functionality of female ovarian follicles and ovulation [1,2]. Naturally, the AR is directly modulated through a ligand-dependent mechanism. In the unliganded state (also called *apo* conformation), AR is anchored into the cytoplasm by heat shock proteins and immunophilins. Upon endogenous ligand binding, i.e., androgen hormones such as testosterone and dihydrotestosterone, AR undergoes conformational changes and dissociates from its complex [3]. In the canonical pathway, also referred to as the genomic pathway, the freed ARs homodimerize and are recognized by importins that ensure their translocation into the nucleus [4]. Inside the nucleus, the homodimer–ligand complex interacts with co-activators and accesses a DNA-exposed androgen response element where they induce the transcription of an ensemble of genes [1,5]. However, a nongenomic pathway also exists at a low androgens concentration in which the AR interacts with substrates from the cytoplasm close to the inner membrane and plays a direct role in kinase cascade activation. This nongenomic signaling action of AR triggers cell proliferation and survival [6].

AR activity is associated with several pathological processes including prostate, testicular and ovarian cancers, impaired reproduction system development and neuromuscular diseases [7]. Thus, AR modulators have been developed and tested for therapeutic purposes. For instance, activation of the AR is responsible for the progression of prostate cancer in patients. AR deprivation therapies were thus developed to treat those patients, whether through surgical orchidectomy up to the 1950s, or through a chemical castration, by administering luteinizing hormone-releasing hormone (LHRH) analogues that block androgen production [8]. The additional administration of AR-antagonist compounds in castration-resistant prostate cancer (CRPC) prolongs the survival of patient in most cases [7].

Another example is the use of AR agonist compounds in the treatment of Duchenne muscular dystrophy (DMD). Indeed, diminished anabolic actions of androgen receptors have been related to rapid health deterioration and shorter lifespan on prepubertal boys with DMD [9,10,11]. Preclinical studies support the importance of AR agonist compounds in improving DMD-related musculoskeletal and cardiopulmonary complications, as well as survival extension on DMD-mouse models [9]. To this aim, nonsteroidal tissue selective androgen receptor modulators (SARMs) are being developed, since they present osteo- and myoanabolic activity, and a reduced growth effect on sexual organs and secondary effects in comparison to steroids compounds [11]. The nongenomic role of AR in neuronal differentiation induced by both androgens and nerve growth factor in PC12 cells as highlighted by Di Donato et al. also provides a potent important role of AR agonist compounds in proliferative or neurodegenerative diseases [12].

More recently, androgen hormones signaling dysregulation caused by unexpected binding of environmental compounds to AR has been pointed out, revealing its involvement in toxicological pathways. Given the central role of AR in ensuring normal muscle, bone, and reproductive organ development and functions, the identification of human exposed chemicals that might mimic endogenous ligands by binding and modulating the AR is of primary interest. This represents a shift; since the aim is no longer to identify drug candidates from chemical libraries acting through AR modulation, but to predict which compounds present in our (daily) environment can be involved in the pathogenesis of several diseases. To gain more insights about these compounds, U.S. federal agencies have recently tested 10K substances in toxicity-related pathways, including AR, under the Toxicology in the 21st Century Program (Tox21). However, the number of environmental compounds suspected to interfere with these toxicity-related pathways is very large and is increasing rapidly. As experimental testing is a long and costly process, the outcomes of the Tox21 program are currently analyzed worldwide to build prediction models that would be crucial to predict, reduce and prioritize the compounds to be experimentally tested.

To build prediction models, either with a therapeutic or toxicological purpose, a large number of *in silico* methods can be used, which are also called virtual screening methods [13]. These methods are mainly classified into two main categories, depending on the input data they require. The first category, called “structure-based” (SB), refers to methods that depend on the availability of an experimentally resolved structure of the target (X-ray crystallography, NMR, cryo-EM). The second category, the “ligand-based” (LB) methods, requires knowledge about compounds able to modulate the query target (experimental binding or activity tests, for example). These two categories of methods use different but complementary input data for building prediction models and thus the prediction ability of the corresponding models can differ. These methods are now widely used in complement to chemical and biological protocols, either to guide the chemical synthesis of new compounds, by directing pharmacomodulation within a chemical series or by identifying potent compounds with new and unexplored scaffolds, or to prioritize the experimental screening of large data sets of molecules.

In this paper, we focus on the prediction of AR agonist compounds using virtual screening methods. The availability of experimentally validated AR-agonist/antagonist and non-binding compounds recorded in the Nuclear Receptor DataBase Including Negative Data (NR-DBIND) [14], and of numerous experimentally elucidated AR structures in an agonist-bound conformation from the Protein Data Bank (PDB), make both LB and SB methods relevant. Therefore, we compared the ability of a pharmacophore modeling (LB) approach and a docking (SB) approach to predict AR agonist compounds, i.e., the ability to discriminate the agonist compounds from antagonist and non-binding compounds. Both approaches have been trained on AR-related NR-DBIND data and tested on an external dataset extracted from the Tox21 [15]. As compared to most *in silico* models proposed previously, those presented in this paper integrate the information of known inactive data, i.e., ligands experimentally tested and that are unable to trigger an intended activity on the query target. We suggest that integrating this information into model construction, evaluation and selection is crucial and should minimize the gap observed between retrospective and prospective performance of prediction models. Herein, we first detail the content of the training set used and in particular, the close structural similarity observed between agonist and antagonist compounds. Then, the performance of pharmacophore models built with LigandScout [16,17] and of two free docking software programs (PLANTS [17] and AutoDock VINA [18]) are evaluated, compared and discussed from both the therapeutic and public health points of view. This study should orient further protocols of AR-agonist compounds investigation in choosing and applying the adapted method depending on the intended hit discovery purpose.

## 2. Materials and Methods

### 2.1. Data Sources

#### 2.1.1. NR-DBIND

Small molecules and protein structures were selected from NR-DBIND (http://nr-dbind.drugdesign.fr/) [14]. This database provides affinity data for small molecules that were experimentally tested against NRs (including negative results) together with pharmacological profile annotations (whenever the information was available) and annotated protein structures. In this study, binders with an agonist pharmacological profile annotation were extracted from the NR-DBIND to constitute the active compounds set. In the same way, binders with an antagonist pharmacological profile annotation and non-binders were extracted and gathered in the inactive compounds set. Ambiguous binders, i.e., binders with no activity annotation and binders with both « agonist » and « antagonist » annotations, were dismissed. In total, the active set contains 224 molecules and the inactive set contains 588 molecules (451 antagonists and 137 non-binders).

#### 2.1.2. Tox21

During the Tox21 high throughput screening (HTS) protocol, agonists of the AR signaling pathway were identified using a beta-lactamase reporter gene cell-based assay and an additional autofluorescence counter-screen assay performed to discard false positive signals. Results of these Tox21 HTS assays were extracted from the PubChem BioAssays platform (AID 743053) [19]. Even though there is no clear evidence of the agonist compounds directly interacting with the AR ligand binding domain (LBD), the high concordance between measured activities and the LBD sequences phylogeny of all tested NRs suggests that the binding to LBD is probably essential for activating the NR [20]. Molecules (i.e., unique CIDs) with agonist activity values <1 µM were included in the active external test set; those presenting “inconclusive” agonist activity were discarded. The molecules that did not show any ability to interact with AR were gathered in the inactive external test set. In total, the active external test set contains 100 molecules (see the list in Table S1) and the inactive external test set contains 5590 molecules.

### 2.2. Small Molecules Preparation

Molecules from the NR-DBIND were extracted in SMILES format, and molecules from the Tox21 were extracted in SDF format. The majority of the protonated microspecies at pH 7.4 of each molecule was computed using Marvin (Version 17.22.0, 2017, ChemAxon, http://www.chemaxon.com). For each compound, 3D conformations were generated using iCon as implemented in LigandScout [16] (Version 4.3.) and only the lowest-energy conformation of each compound was considered. MGLTools was used to convert ligand SDF files into the AutoDock Vina PDBQT format by assigning Gasteiger charges and atom types.

### 2.3. Molecule Similarity Networks and Chemical Space Analysis

The molecules of the active and inactive compounds set extracted from the NR-DBIND were translated into MACCS fingerprints. These fingerprints describe each molecule as a 166-bit string of 1 and 0 values, indicating the presence or the absence of a given chemical group. Pairwise structural comparisons were computed through Tanimoto coefficient calculation between two MACCS fingerprints using RDKIT [21]. Cytoscape [22] was used to graphically display networks of structurally similar molecules. In this representation, nodes symbolize the small molecules, and edges are connections between two small molecules sharing a Tanimoto coefficient above a given threshold. In a second approach, classical descriptors were computed for each molecule (i.e., molecular weight, cLogP, number of H-bond donors, number of H-bond acceptors, total surface area, total polar surface area, MolFlex, MolComplex, number of rotatable bonds, number of aromatic rings, number of aromatic atoms). These descriptors were used to study the chemical space covered by each dataset using a principal component analysis achieved with the software DataWarrior.

### 2.4. Protein Preparation

Following protein structure selection guidelines [23] on the impact of the pharmacological profile of the co-crystallized ligand on docking performances, only AR agonist-bound structures listed in the selected NR-DBIND were considered and downloaded from the Protein Data Bank (PDB) [24] (Table S2). All structures (29 in total) were protonated at pH 7.4 with PDB2PQR (Version 2.1.1). Water molecules and heteroatoms were removed.

### 2.5. Pharmacophore Modelling Protocol

#### 2.5.1. Primary Pharmacophore Modelling

NR-DBIND affinity data are expressed either in pKi or in pIC_50_. Since these data are not directly comparable, they were considered separately for pharmacophore modeling, i.e., pKi-pharmacophores were generated using pKi data and pIC_50_-pharmacophores using pIC_50_ data. For each subset (pKi and pIC_50_), active and inactive molecules were divided into training (~2/3) and test sets (~1/3) using 25 random sampling with replacement. For each subset, we thus obtained 25 pairs of training and test sets and the 25 training sets were used as a starting point for pharmacophore generation. Pharmacophore models were generated with LigandScout 4.3 [16,17] as follows:

1. The training set molecules were clustered using LigandScout default parameters;
2. “Merged” pharmacophore models were generated, i.e., models considering features common to at least 10% of the cluster small molecules. 10 pharmacophore models were generated per cluster.

#### 2.5.2. Pharmacophore Selection

Active and inactive molecules from the training sets were screened into each corresponding pharmacophore models authorizing up to 50 omitted features. A pharmacophore matching score was attributed to each screened molecule, accounting for the number of matched features and the coverage of the pharmacophore features as compared to the reference model. This score is indirectly linked to the number of omitted features: e.g., a molecule A matching more pharmacophore features than a molecule B will be automatically attributed a better score. Therefore, sensibilities and specificities could be computed for different score thresholds and allowed determining the ideal number of pharmacophores features than can be omitted to reach high specificity. This information was used to determine the starting point for the pharmacophore optimization (cf. Section 2.5.3.). Performance of the pharmacophore models were quantified in terms of sensibility, specificity and enrichment factors at 25%. The sensibility quantifies the ability of a model to retrieve positive data (i.e., agonist compounds) classified as such:

(1) $$\mathrm{Sensibility}=\;\frac{\mathrm{Number}\;\mathrm{of}\;\mathrm{True}\;\mathrm{Positives}}{\mathrm{Number}\;\mathrm{of}\;\mathrm{True}\;\mathrm{Positives}+\;\mathrm{Number}\;\mathrm{of}\;\mathrm{False}\;\mathrm{Negatives}},$$

The specificity quantifies the ability of a model to correctly reject negative data (i.e., antagonist compounds and non-binders):

(2) $$\mathrm{Specificity}=\;\frac{\mathrm{Number}\;\mathrm{of}\;\mathrm{True}\;\mathrm{Negatives}}{\mathrm{Number}\;\mathrm{of}\;\mathrm{True}\;\mathrm{Negatives}+\;\mathrm{Number}\;\mathrm{of}\;\mathrm{False}\;\mathrm{False}\;\mathrm{Positives}}$$

The enrichment in agonist compounds in the first 25% of the ordered screened database according to the pharmacophore matching score (EF_25%_), was computed as follows:

(3) $${\mathrm{EF}}_{25\%}=\frac{{\mathrm{Number}\;\mathrm{of}\;\mathrm{Actives}}_{25\%}/{\mathrm{Totale}\;\mathrm{Number}\;\mathrm{of}\;\mathrm{Molecules}}_{25\%}}{{\mathrm{Number}\;\mathrm{of}\;\mathrm{Actives}}_{100\%}/{\mathrm{Totale}\;\mathrm{Number}\;\mathrm{of}\;\mathrm{Molecules}}_{100\%}}$$

To select pharmacophore models that should be further optimized, all the models generated with the 25 training sets (generated from the 25 random sampling procedures) were ranked according to their EF_25%_ values in descending order, with a prioritization of models that retrieved structurally unrelated molecules in case of very similar EF_25%_ values. According to this ranking, one training set was selected for each of the pKi and the pIC_50_ subsets—the one associated to the higher number of top-ranked pharmacophore models; the others were discarded from the study. The pKi and pIC_50_ pharmacophore models associated with the selected training set were optimized until no improvement in the sensibility nor selectivity was measured.

#### 2.5.3. Pharmacophore Models Optimization

The pharmacophore optimization protocol consists of two steps:

Active and inactive molecules from the training sets were screened with a decreasing number of allowed omitted features. This step was repeated as long as at least two Bemis–Murcko scaffolds from the active compounds set were screened and until the number of screened inactive molecules was null or stopped decreasing.

The pharmacophore model was modified to improve the sensibility of the model (screening of a maximum of agonist compounds) while controlling its specificity (discarding a maximum of inactive compounds): exclusion volumes were added, some features position slightly displaced, and some tolerance sphere and weight were modified through visual learning. A modification leading to the screening of a new Bemis–Murcko scaffold was accepted despite the screening of a few (up to five) additional inactive molecules.

The sensibility, the specificity and the enrichment factor were computed on the training and the test set for each ensemble of retained pharmacophore models (pKi and pIC50); pKi set derived models were tested on the pIC50 set, and conversely, pIC50 set derived pharmacophore models were tested on pKi set. Finally, both ensembles of pharmacophore models were tested on the Tox21 external set.

### 2.6. Small Molecules Docking Protocol

The docking of the NR-DBIND extracted molecules was performed with two software programs with free academic licensing: AutoDock Vina 1.1.2 [18] and PLANTS. AutoDock Vina generates docking poses using an iterated local search for global optimization, which consists in a succession of stochastic mutations steps and local optimizations. PLANTS uses an ant colony optimization algorithm that treats small molecules and hydrogen atoms of residue side chains with flexibility. Docking was performed on the 29 AR agonist-bond structures. Consideration of a single structure may impair the docking performance either if the selected structure is not adapted to the study (marginal conformation, closed conformation, unusual ligand-bound study, etc.), or if the protein presents local to global flexibility, which must be considered [25]. We thus analyzed and compared the docking results obtained with single structures and ensembles of two and three structures, with the aim to identify the best docking protocol. For both single structure and ensemble docking, the top score of each ligand was considered. Performances were evaluated in terms of area under the ROC curve (AUCs), specificity and sensibility (cf. equations (1,2)). The probability of activity at a given score was estimated via the online Screening Explorer [26].

The structures yielding the best AUCs on the NR-DBIND set for each docking program and each scenario ((1) single structure, (2) two structures and (3) three structures ensemble docking) were used for external validation using the Tox21 data.

## 3. Results

### 3.1. Similarity Between Agonist and Non-agonist Compounds

**Figure 1:**
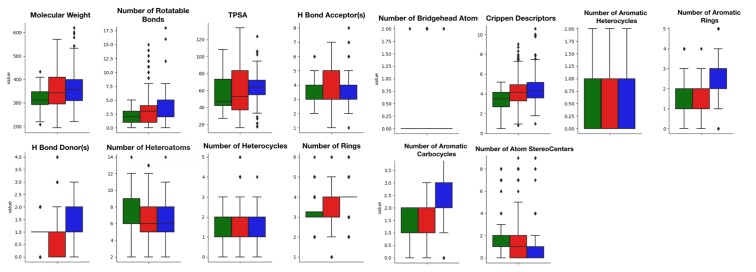
Boxplots of descriptor values computed with RDKIT on agonist compounds (green), antagonist compounds (red) and non-binders (blue).

Common structural descriptors (molecular weight, number of rotatable bonds, total polar surface area, number of H-bond donor, number of H-bond acceptor, number of bridgehead atoms, Crippen descriptor (logP), number of aromatic heterocycles, number of aromatic rings, number of heteroatoms, number of heterocycles, number of rings, number of aromatic carbocycles and number of atom stereocenters) were computed to compare the overall similarity between agonist compounds from the active set and antagonist compounds and non-binders from the inactive set (Figure 1).

**Figure 2:**
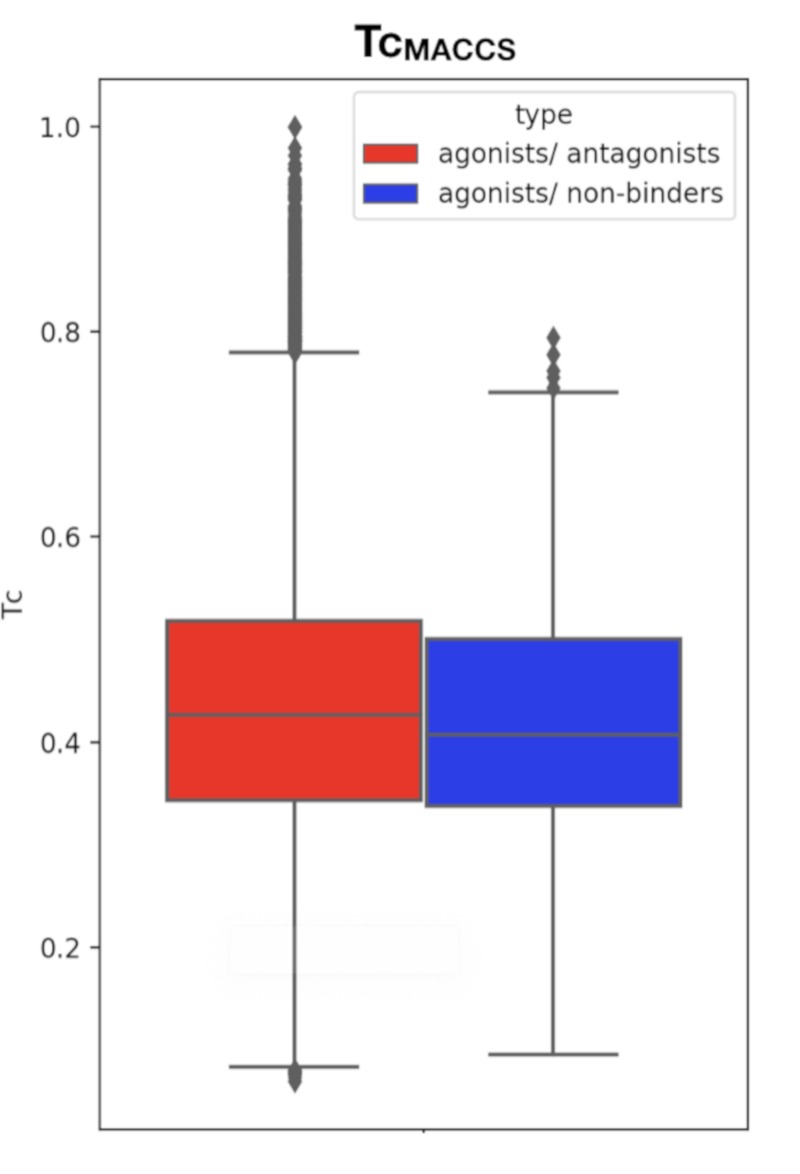
Boxplot of the Tc_MACCS_ values computed between agonist and antagonist compounds (left, red) and agonist and non-binder compounds (right, blue).

**Figure 3:**
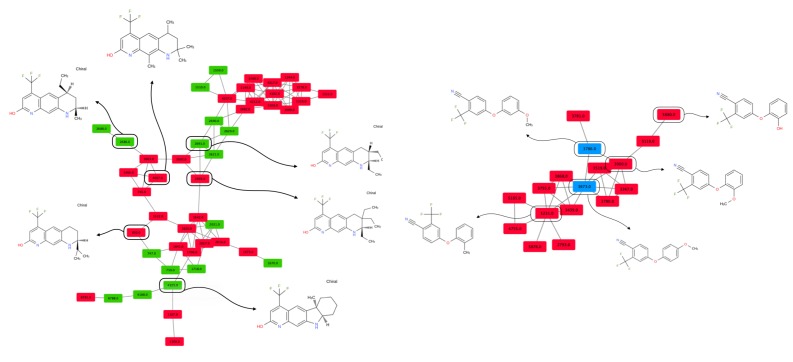
Molecular networks of molecules sharing structural similarity. Colored rounded rectangles represent molecules (green: agonist, red: antagonist, blue: non-binder). Each edge connects two molecules with a Tc ≥ 0.90.

This comparison is necessary to ensure that the molecules from the active and the inactive sets cannot be discriminated solely on the basis of physicochemical properties. Results show that agonist and antagonist compounds present similarities regarding the number of aromatic heterocycles, the number of aromatic rings, the number of heterocycles and the number of aromatic carbocycles. They also share similarities with the non-binders in terms of number of H-bond acceptors, number of aromatic heterocycles and number of heterocycles. A remarkable output of these comparisons is that most agonist compounds have a single H-bond donor (78% of the agonist compounds, 36% of the antagonist compounds and 52% of the inactive compounds), while most antagonist compounds have from 0 to 2, and non-binders have up to 3 H-bond donors. Agonist compounds also present an overall smaller heavy atom molecular weight (HMW), a smaller number of rotatable bonds and a very steady number of rings (3) as compared to compounds from the inactive set. The pairwise structural similarity was computed as the Tanimoto coefficient (Tc) between the MACCS representation of molecules composing the active and the inactive set. Again, a higher similarity is observed between the agonist and the antagonist compounds than between agonist and non-binder compounds. We also observe that a non-negligible number of molecules from the inactive set share Tc > 0.7 with the agonist compounds (Figure 2). Mapping a molecular network illustrating the connections between molecules sharing Tc > 0.9 (Figure S1) highlights high scaffold similarities between some agonist and antagonist compounds, and some agonist compounds and non-binders (Figure 3). The chemical space was analyzed via a principal component analysis based on 11 physicochemical descriptors and confirmed a good coverage of the active and inactive sets (Figure S2).

### 3.2. Pharmacophores Ability to Discriminate Agonist Compounds

#### 3.2.1. Performance on the NR-DBIND Datasets

The selected and optimized pharmacophores (cf. 1.2.5) were built using two training sets (pKi and pIC_50_) containing, respectively, 89 and 58 compounds in the active set and 182 and 216 compounds in the inactive set. The corresponding test sets are respectively composed of 48 and 34 compounds in the active set and 90 and 107 compounds in the inactive set. In total, nine pharmacophore models were derived from the pKi set and referred as pKi-pharmacophores, and nine other pharmacophore models were derived from the pIC50 set and referred to as pIC_50_-pharmacophores. Sensibilities, specificities and EF obtained through pKi- and pIC_50_-pharmacophores screening are summarized Table 1. Both ensembles of pharmacophores reach high performances in retrieving active compounds (sensibility > = 0.94) and in discarding inactive compounds (specificity > = 0.88) on their respective training set. The sensibility slightly drops on their respective test set while the specificity remains steady. The pKi-pharmacophores screening of the pIC_50_ set displays lower performances (sensibility = 0.61, specificity = 0.62) and pIC_50_-pharmacophores screening of the pKi set reveals low sensibility (= 0.23) while conserving the ability of discarding inactive compounds (specificity = 0.87). The screening on the entire NR-DBIND set (training and test sets melted) with both pKi- and pIC_50_-pharmacophores reveals a high global sensibility (= 0.92) and specificity (= 0.68).

#### 3.2.2. Performance on the Tox21 Dataset

The Tox21 set was screened with pKi- and pIC_50_-pharmacophores. The Tox21 set is composed of 5590 inactive compounds and a varying number of active compounds according to the query activity threshold (< 1 µM = 100; < 100 nM = 70 < 10 nM = 29). The pKi-pharmacophores screening displays very low performance in retrieving active compounds (sensibility = 0.01), while the pIC_50_-pharmacophores allow retrieval for some of them (sensibilities from 0.24 (< 1µM and < 10 nM) to 0.29 (< 100 nM)). Screening with both ensembles of pharmacophores (separately) present high specificity on the Tox21 dataset (> 0.90). Combining pKi- and pIC_50_-pharmacophores results in similar outcomes as those obtained with the pIC_50_-pharmacophores alone. The specificity reached is similar to the one obtained with NR-DBIND data; nonetheless, the sensibility is considerably lower (sensibility_Tox21_ <1 µM = 0.24, Δspecificity = −0.68). To provide a rationale for this observation, the chemical space covered by the agonist compounds from the Tox21 and the agonist compounds from the NR-DBIND were compared. The results highlight the poor chemical space overlap between the two sets (Figure S2).

### 3.3. Docking Performances

#### 3.3.1. Performance on the Training Set

**Figure 4:**
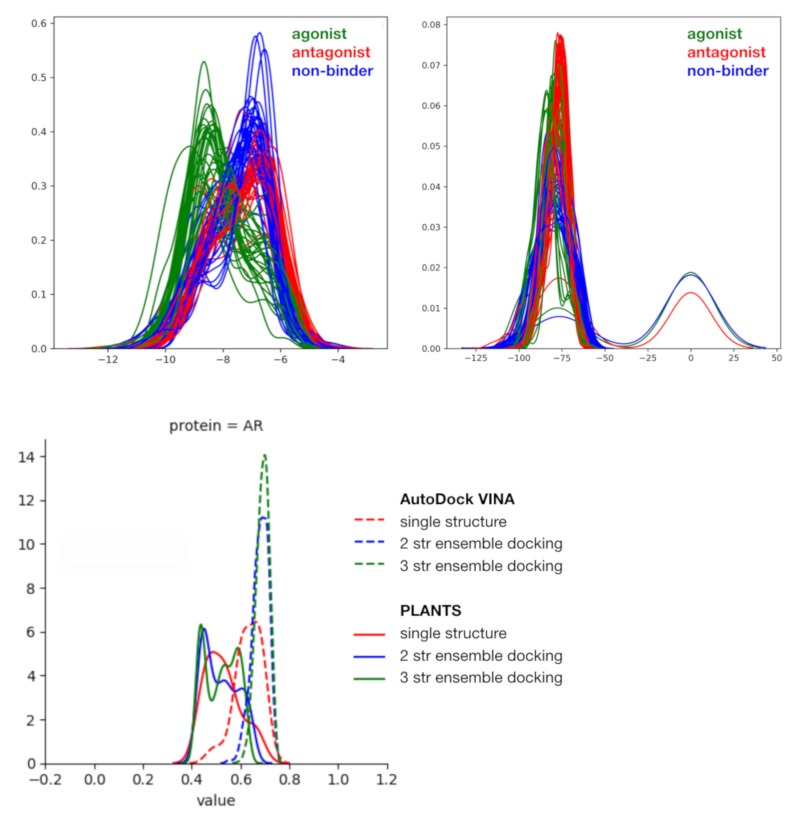
Illustration of the docking results; score distributions (upper panels) obtained with AutoDock VINA (left) and PLANTS (right); distributions of AUCs obtained with each docking scenario (tools: AutoDock VINA and PLANTS, docking type: single structure docking, two- and three-structures ensemble docking).

In order to identify the best docking protocol for the prediction of AR agonist compounds, we performed the docking of the whole pKi-pIC_50_ dataset with two different open access software programs, AutoDock VINA and PLANTS, on AR agonist-bound PDB structures. In a more global study (unpublished), we showed that the performance of the two docking software programs vary according to the studied nuclear receptors; thus, none of them should be systematically preferred over the other. A detailed analysis of the docking results is necessary to identify the protocol that is best suited for the agonist-compounds prediction model. Here, the score distributions obtained for each compounds type (agonist/ antagonist/ non-binder) reveal that AutoDock VINA outperforms PLANTS in discriminating agonist compounds (active set) from the others (inactive set) (Figure 4). AUCs were computed for each single structure and each ensemble of two and three structures (Figure 4 and Table 2). AutoDock VINA yields higher AUC performance on this system as compared to PLANTS. Using AutoDock VINA, the distribution of AUCs values associated with the two- and three-structures ensemble docking outcomes is shifted toward high values with higher maximal, mean, and minimum values and smaller standard deviation than single structure docking. This trend is not observed with PLANTS. Even though AutoDock VINA performances improve with the number of structures, the difference between two- and three-structures docking results remains small and does not highlight the need for considering larger ensemble of structures. The combination of PLANTS and AutoDock VINA into consensus rankings as proposed by Screening Explorer [26] does not improve the observed performances, except for the single docking structure that reaches an AUC of 0.754, considering the average ranking.

The pharmacophore models mentioned previously allow for a binary classification of the compounds. At the opposite, using a docking method, continuous scores are attributed to docked poses and can serve as a ranking criteria. However, it is possible to define thresholds to transform the continuous score into a binary classifier and deduce a specificity and a sensibility from it. Depending on the screening purpose, it might be of interest either to reach a certain sensibility (capability of retrieving the active compounds) or a high enrichment factor (EF) to ensure a high probability of having active compounds in a screened fraction (P (Active)). Thus, we investigated 1) the scores, specificities and EF reached at fixed sensibility values (0.25/ 0.5/ 0.75–Table 3) and 2) the scores, specificities, sensibilities and the number of active compounds retrieved at fixed P (Active) (0.25/0.5/0.75–Table 4). The observed scores are indicatives and might be reused for prospective studies on blind datasets. As expected, the specificity considerably drops with an increasing sensibility. Different threshold scores around −90 for PLANTS and −9.20 for AutoDock VINA enable reaching P (Active) = 0.5, i.e., the probability of having 50% of active compounds. AutoDock VINA results could help defining threshold scores to reach P(Active) = 0.66 and 0.75 as summarized in Table 4.

#### 3.3.2. Performance on the Tox21 Set

The performance of AutoDock VINA and PLANTS was evaluated using an external dataset extracted from the Tox21 screening campaign results. Docking was performed on each structure or ensemble of structures previously associated with the best AUC with the pKi and the pIC_50_ data set, respectively. All AUCs obtained exceed that obtained with the NR-DBIND set, excepted for the single structure docking with PLANTS (Table 5). Again, AutoDock VINA outperforms PLANTS on this dataset, but the ensemble docking approach is not associated with enhanced overall performance. High specificities (0.99) are observed for a sensibility fixed at 0.25 with AutoDock VINA for every scenario, and consequently, very high EF are obtained (>15).

### 3.4. Pharmacophore Modeling vs. Docking on the NR-DBIND Set

**Figure 5:**
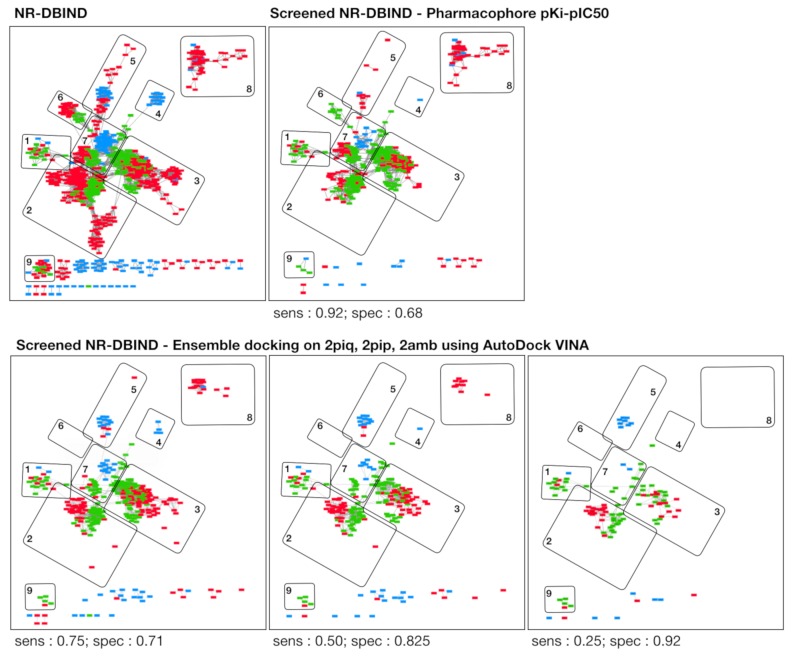
Networks of structurally similar agonist compounds (green), antagonist compounds (red) and non-binders (blue). Rectangles correspond to molecules, and two molecules are connected by and edge when their pairwise Tc ≥ 0.7. The global network is represented in the top left panel (NR-DBIND), and the other panels represent the remaining compounds after screening using the pharmacophore approach (pharmacophore pKi-pIC_50_, top-right panel) and using the docking approach (ensemble of three structures, 2piq, 2pip, 2amb, bottom panels).

We compared the performance obtained with respectively the pharmacophore modeling approach and the best docking approach (AutoDock Vina, ensemble of three structures). The best docking performance was evaluated at fixed sensibility and specificity values to be compared with the pharmacophore modeling approach (sensibility = 0.92 and specificity = 0.68). Remarkably, specificity obtained with the docking approach at sensibility = 0.92 is lower than the specificity obtained with the pharmacophore modeling approach (specificity_docking_ = 0.457; Δspecificity = −0.223). The same trend is observed for the sensibility associated with the docking approach for a specificity set to 0.68 (specificity_docking_ = 0.781; Δsensibility = −0.139). Nonetheless, the docking approach enables reaching higher specificities than the pharmacophore approach, to the disadvantage of sensibility that consequently decreases. We compared the aforementioned similarity network at Tc ≥ 0.7. The molecules screened with the pharmacophore approach and the three-structure ensemble docking (pdb: 2piq, 2pip, 2amb) with AutoDock VINA are compared to the unscreened networks. We observed that the use of inactive molecules for pharmacophore optimization enables rejecting molecules strongly embedded into the network (Figure 5—zone 2/3/5/6), but not some of the inactive molecules structurally unrelated to the active set molecules, which also happen to be absent from the training set (Figure 5—zone 8). The docking approach seems better fitted for nontrained scaffold screening (Figure 5—zone 8) but hardly discriminates active compounds from inactive compounds in highly connected zones (Figure 5—zone 2/3/7). Interestingly, both the pharmacophore and the docking approach allow retrieving active molecules from every defined zone (except for zone 6 for the docking approach).

## 4. Discussion

### 4.1. NRs Modulators’ Tricky Structures

In agreement with the literature [27,28], the analysis of the NR-DBIND dataset reveals high structural similarities between some agonist and antagonist compounds. So far, despite the attempts to build agonism/ antagonism prediction tools [28,29,30], the frontier between the activity of agonist and antagonist ligands remains unclear. Lagarde et al. have generated 3D agonist and antagonist pharmacophores that are selective for the NRLiSt BDB agonist and antagonist ligands chemical space [31]. To reach selectivity for agonist and antagonist compounds, up to 52 and 64 structure-based and ligand-based pharmacophores per NR were respectively necessary, highlighting the difficulties to understand their binding modes. Interestingly, physicochemical differences were depicted between the agonist and antagonist selective pharmacophores with agonist pharmacophores displaying significantly less hydrogen bond acceptor, hydrophobic, aromatic ring, positive ionizable and negative ionizable features than the corresponding antagonist selective pharmacophores for respectively 9, 5, 4, 2 and 1 NRs. Our descriptors analysis is in agreement with those observations since the studied agonist compounds globally present smaller HMW, fewer rings and fewer HBD than both antagonist compounds and non-binders. More specifically, they have a unique HBA that is likely to play a key role in the compounds binding and target modulation. Resolved 3D structures of NRs in complex with different compounds should help understanding the structural determinants of NRs ligands leading to agonism or antagonism. At a microscopic scale, the *apo* and *holo* X-ray structures of NRs have revealed that triggering the agonist or antagonist activity is tightly related to the helix H12 placement. In an unbound state, the helix H12 is displaced from the LBD, exposing the co-repressor binding site [32,33,34,35]. The binding of an agonist ligand leads to H12 packing against the LBD, forming a buried binding site, and occluding the co-repressor binding site while structuring the co-activator binding site. It is now known that antagonist ligands prevent coactivator binding through perturbation of the H12 packing but the molecular mechanisms controlling the antagonist activity are less understood. One hypothesis is that antagonist compounds possess a bulky extension that dislodges H12 from the LBD. However, the LBD adaptation to those bulky extensions does not systematically impact the stability of H12; therefore, depending on the nature and the size of the extension, can induce activities from agonism to antagonism [28]. Another hypothesis is that some antagonist compounds function in a passive manner through a lack of specific contacts with the LBD, thus perturbing the helix H11 and indirectly, the H12 [33]. Finally, some compounds have a dual action, i.e., they elicit an agonist activity in some tissue, and an antagonist activity in others.

### 4.2. Recommendations on Screening Strategies

Herein, we present two *in silico* screening protocols to predict AR agonist compounds. To do so, we evaluated the performance of the protocol in discriminating agonist compounds of the AR from non-agonist compounds (antagonist compounds and non-binders). The two approaches detailed in this paper consist in a pharmacophore modeling (LB) and a docking (SB) approach and both constitute suitable strategies for screening AR agonist compounds. However, results differ in term of sensibility and specificity and should be carefully analyzed to rationally favor one approach over the other, depending on the intended hit discovery purpose. Therapeutic research tends to favor enrichment of active compounds in the top ranked compounds in order to achieve good hit rates while minimizing the number of required experimental assays, favoring high specificity values. At the opposite, prediction of undesired interactions, either for side effect prediction, for toxicologic assessment or for identifying potent endocrine disrupting compounds necessitates maximizing the sensibility of the screening to identify a maximum of potentially undesired compounds. Of note, both sensibility and specificity terms are considered for therapeutic, toxicology and public health research topics, and everything is a question of balance.

Herein, the pharmacophore approach is displaying the highest sensibility (= 0.92) at a correct specificity value (= 0.68) on the NR-DBIND data. Due to the use of inactive data information during the training phase, this approach has the outstanding capacity of discriminating between active and inactive compounds that are structurally related, which is less the case with the docking approach (cf. Figure 5). Nonetheless, the same models applied on the Tox21 dataset maintain a good specificity (= 0.88) but display a lower sensibility (0.24 for the < 1 µM and <10nM subsets to 0.29 for the < 100nM dataset). The drop in sensibility observed when applying the model on the Tox21 data is mostly explained by the low chemical space overlapping between agonist compounds from the NR-DBIND and the Tox21 datasets (Figure S2). The poor applicability of pharmacophore models on a dataset unrelated to the training set limits its use for undesired effect prediction and therapeutic research. We suggest that the publication of more active and inactive data should tend to alleviate this trend.

Regarding the docking approach, AutoDock VINA outperforms PLANTS in discriminating AR agonist compounds and presents better AUCs output when considering ensembles of two or three structures rather than a single structure (0.66 ± 0.05/0.7 ± 0.03/0.74 ± 0.02 for AutoDock VINA and 0.56 ± 0.06/0.56 ± 0.06/0.56 ± 0.05 for PLANTS (order: 1 str/2 str/3 str)). Structures associated with the best AUCs for each docking software were retained for further analysis and used to screen the Tox21 external test set with the docking approach. High AUCs values (0.65/0.78/0.79 with PLANTS and 0.82/0.8/0.81 with AutoDock VINA) were obtained in this external validation step. As previously observed with the NR-DBIND set, AutoDock VINA was more adapted for the studied system and no performance improvement was observed with the ensemble docking. Particularly, the high enrichment (>15) observed for sensibilities set at 0.25 with AutoDock VINA encourages using the corresponding structures (2pir, 1t5z/2piw, 2piq/2pib/2amb) together with this software for therapeutic research or to prioritize some compounds for further toxicologic tests in a public health context. However, the low specificity associated with the good sensibilities impairs its applicability to the prediction of larger specter undesired effects. Herein, we have identified threshold scores per software and per docking strategy (single docking, two-structures and three-structures ensemble docking), corresponding to the probability of having 50% of active compounds or more. The application of those thresholds on the Tox21 data yield satisfying results with an average of 31% of actives compounds in the corresponding fraction no matter the method and the intended P(Active) (0.5/0.66/0.75). These thresholds and the models presented here can be applied to identify new agonist scaffolds that could either guide the chemical synthesis of new agonist compounds or to predict the interaction of some existing compounds with AR. The docking protocol can be applied as described with the retained docking protocol (AutoDock VINA with an ensemble of at least two structures, favoring the following ensemble: 2piq, 2pip and 2amb) and default parameters, while the coordinates of the retained pharmacophore models are given in Table S3.

### 4.3. Publication Bias

In the present study, agonist molecules constitute the active set and are opposed to both antagonist molecules (224 molecules) and experimentally validated non-binders that constitute the inactive set (588 molecules). The choice of including only experimentally validated non-binders and not, like many other databases, putative inactive compounds (the so called decoys) confront us with unusually high active/inactive molecules ratio (~1/2.6 as compared to 1/50 in the gold standard benchmarking data base, the DUD-E [34]). Ideally, active/inactive compounds ratio should be close to those observed in real life HTS experiments, with a hit rate success estimated around 0.1 to 4% [35]. Considering datasets with lower ratio should strengthen the reliability and the transposability of the generated/selected models. However, that data is scarce, and we deplore the lack of inactive data publication (for example, as supplementary online material in publications). Of note, valuable information can be brought from inactive compounds extracted from chemotypes that were successfully derived into hits/leads but also from chemotypes that resulted in a complete failure. In comparison to the decoys that bring no information to the real specificity of the models, the use of experimentally validated inactive data provides this information for the chemical space they cover. We suggest that the massive publication of inactive data, so far considered as failure, should be encouraged. The PubChem Bioassay is an example of robust database for reliable model construction based on exclusively experimental data; this database gathers over 230 million HTS bioactivity records provided by academic and private institutes that include all tested molecules, including screened inactive compounds. However, HTS data still suffers from a lack of quality control. Despite false positive identification through autofluorescence and other HTS-material interfering assays, no study has quantified the risk for false negatives in primary assays as of yet. We believe that when more quality data is published, human- or material-related errors in the models’ construction and evaluation will be minimized.

## 5. Conclusions

AR is a nuclear receptor involved in many biological processes. It can be modulated by exogenous ligands, whether intentionally with drugs to treat diseases such as prostate cancer, or unintentionally by environmental contaminants. Thus, predicting the ability of a compound to interact with AR is of major interest for both drug and toxicity prediction. Prediction can be achieved using *in silico* methods, for which publication of bioactivity, affinity and structural data constitute the critical basis for accurate prediction performance. In this paper, we evaluated and compared the ability of two screening strategies (docking and pharmacophore modeling approaches) to discriminate AR agonist compounds from a set of inactive data encompassing both experimentally validated AR antagonist and non-binding compounds. We first studied the structural similarity between the agonist and the inactive data sets and showed that some compounds present high structural similarities. These compounds represent a real challenge for prediction models. The pharmacophore models-based strategy appears to be particularly suited to discriminate the agonist compounds from the inactive set, and is especially effective to discriminate structurally similar agonist and antagonist compounds. Nonetheless, its lower performance on an external dataset in terms of sensibility underlines the necessity to have diversity both in the active and the inactive set to build models applicable to a large chemical space. The prediction quality of pharmacophore models for both undesired effect prediction and therapeutic research should significantly benefit from massive data publication. The high specificity conserved on the external dataset makes it suitable for therapeutic research. The docking tool AutoDock VINA also appears particularly suited for AR agonist prediction, particularly when applied on two or three agonist-bound structures. The method is fitted to enrich the screened database in agonist compounds and the parameters proposed here should help to conduct successful docking studies for therapeutic purposes. In addition, scores corresponding to balanced specificity/sensibility and EF are proposed and should help biologists to select potent AR agonist compounds for further testing in a prospective study.

## Figures and Tables

**Table 1:** Performances of the pKi- and pIC_50_-pharmacophores screening on the NR-DBIND and the Tox21 datasets in terms of sensibility (sens), specificity (spec) and enrichment factor (EF).

	Set	pKi-Pharmacophores			pIC_50_-Pharmacophores			pKi-pIC50-Pharmacophores		
		Sens	Spec	EF	Sens	Spec	EF	Sens	Spec	EF
**NR-DBIND**	pKi	Train: 0.95 Test: 0.80	Train: 0.88 Test: 0.89	Train: 2.44 Test: 2.03	0.23	0.87	1.22	-	-	-
	pIC50	0.61	0.62	1.43	Train: 0.98 Test: 0.74	Train: 0.94 Test: 0.91	Train: 3.45 Test: 3.08	-	-	-
	pKi-pIC50	0.78	0.73	1.90	0.53	0.89	2.33	0.92	0.68	1.88
**Tox21**	Tox21 (<1 µM)	0.01	0.90	0.06	0.23	0.95	2.53	0.24	0.88	1.15
	Tox21 (<100 nM)	0.01	0.90	0.09	0.27	0.95	3.47	0.29	0.88	1.57
	Tox21 (<10 nM)	0.00	0.90	0.00	0.24	0.95	3.44	0.24	0.88	1.45

**Table 2:** Details of AUC distributions obtained with PLANTS and AutoDock VINA.

		PLANTS					AutoDock VINA				
		Max	Pdb	Min	Mean	Std	Max	Pdb	Min	Mean	Std
AR	Single	0.69	1xow	0.43	0.56	0.06	0.72	2pir	0.52	0.66	0.05
	ensemble_2	0.69	1xow, 2ama	0.47	0.56	0.06	0.76	1t5z, 2piw	0.6	0.71	0.03
	ensemble_3	0.68	1xow, 1xj7, 2ama	0.46	0.56	0.05	0.78	2piq, 2pip, 2amb	0.64	0.73	0.02

**Table 3:** Specificity, score and EF obtained for each docking scenario on the NR-DBIND dataset at fixed sensibilities (sensibility = 0.25; 0.5 and 0.75).

	PLANTS					AutoDock VINA				
	Max	pdb	sens = 0.25 score spec EF	sens = 0.5 score spec EF	sens = 0.75 score spec EF	max	PDB	sens = 0.25 score spec EF	sens = 0.5 score spec EF	sens = 0.75 score spec EF
Single	0.69	1xow	−89.29	−84.88	−81.36	0.72	2pir	−9.20	−8.7	−8.20
			0.901	0.757	0.578			0.913	0.770	0.611
			1.78	1.59	1.46			1.90	1.64	1.53
ensemble_2	0.69	1xow, 2ama	−90.03	−85.38	−81.70	0.76	1t5z, 2piw	−9.10	−8.6	−8.10
			0.898	0.741	0.548			0.932	0.813	0.646
			1.75	1.54	1.40			2.11	1.83	1.62
ensemble_3	0.68	1xow, 1xj7, 2ama	−90.54	−85.98	−81.81	0.78	2piq, 2pip, 2amb	−9.40	−8.9	−8.5
			0.891	0.741	0.532			0.923	0.825	0.707
			1.69	1.54	1.37			2.01	1.89	1.79

**Table 4:** Score, sensibility, specificity and number of retrieved active compounds at a fixed P (Active) that represents the probability of retrieving active compounds.

	PLANTS				AutoDock VINA			
	Pdb	P (Active) = 0.5 score sens-spec NR-DBIND Tox21	P (Active) = 0.66 score sens-spec NR-DBIND Tox21	P (Active) = 0.75 score sens-spec NR-DBIND Tox21	PDB	P (Active) = 0.5 score sens-spec NR-DBIND Tox21	P (Active) = 0.66 score sens-spec NR-DBIND Tox21	P (Active) = 0.75 score sens-spec NR-DBIND Tox21
Single	1xow	−93.66 0.129–0.963 26 actives	-	-	2pir	−9.50 0.170–0.93939 act/ 36 ina 22 act/ 49 ina	−10.30 0.009–0.9916 act/ 7 ina 6 act/ 9 ina	-
ensemble_2	1xow, 2ama	−93.92 0.138–0.959 32 actives	-	-	1t5z, 2piw	−9.1 0.290–0.918 65 act/ 49 ina 28 act/ 75 ina	−9.8 0.058–0.981 13 act/ 12 ina 15 act/ 25 ina	−10.30 0.027–0.995 7 act/ 3 ina 7 act/ 14 ina
ensemble_3	1xow, 1xj7, 2ama	−94.71 0.116–0.964 26 actives	-	-	2piq, 2pip, 2amb	−9.40 0.277–0.915 62 act/ 51 ina 25 act/ 69 ina	−10.0 0.085–0.981 19 act/ 12 ina 17 act/ 34 ina	−10.40 0.022–0.990 7 act/ 6 ina 4 act/ 17 ina

**Table 5:** Specificity, score and EF obtained for each docking scenario on the Tox21 dataset at fixed sensibilities (sensibility = 0.25; 0.5 and 0.75).

	PLANTS					AutoDock VINA				
	AUC <1µM (<100 nM/ <10 nM)	pdb	sens = 0.25 score spec EF	sens = 0.5 score spec EF	sens = 0.75 score spec EF	AUC <1µM (<100 nM/<10 nM)	PDB	sens = 0.25 score spec EF	sens = 0.5 score spec EF	sens = 0.75 score spec EF
Single	0.65 (0.66/0.7)	**1xow**	−79.38 0.77 1.08	−74.96 0.645 1.4	−70.55 0.520 1.55	0.82 (0.83/0.89)	2pir	−9.4 0.99 17.13	−8.2 0.92 5.72	−7.00 0.7 2.27
ensemble_2	0.78 (0.8/0.85)	1xow, 2ama	−87.97 0.9 12.6	−83.36 0.83 2.8	−77.33 0.69 2.39	0.8 (0.82/0.89)	1t5z, 2piw	−9.3 0.99 19.22	−7.2 0.81 2.55	−6.5 0.63 2.00
ensemble_3	0.79 (0.81/0.84)	1xow, 1xj7, 2ama	−88.29 0.9 2.43	−84.32 0.83 2.9	−78.59 0.715 2.56	0.81 (0.84/0.90)	2piq, 2pip, 2amb	−9.4 0.99 15.98	−8.00 0.88 3.87	7.00 0.680 2.29

## Supplementary Materials

The following are available online at https://www.mdpi.com/2073-4409/8/11/1431/s1, Table S1: Unambiguous Tox21 agonist molecules considered in the study, Table S2: PDB structures used for docking, Figure S1: Structural Network Analysis, Figure S2: Chemical Space Analysis (PCA) of the NR-DBIND and Tox21 datasets, Table S3: Pharmacophore model coordinates.
