# Supplementary material for: Hits Discovery on the Androgen Receptor: In Silico Approaches to Identify Agonist Compounds
Source: Cells. 2019 Nov 13;8(11):1431. doi: 10.3390/cells8111431 (PMC6912550; doi:10.3390/cells8111431)
Supplement: Supplementary file 1 [file cells-08-01431-s001.pdf]

Article

Hits discovery on the Androgen receptor: *in silico* approaches to identify agonists compounds

Manon Réau [1], Nathalie Lagarde [1], Jean-François Zagury [1] and Matthieu Montes [1,*]

[1] Laboratoire GBCM, EA 7528, Conservatoire National des Arts et Métiers, F-75003 Paris, France.;
* Correspondence: matthieu.montes @cnam.fr;

Supplementary Information

List of Content

Table S1. Unambiguous Tox21 agonist molecules considered in the study 2
Table S2. PDB structures used for docking... 6
Figure S1. Structural Network Analysis 8
Figure S2. Chemical Space Analysis (PCA) of the NR-DBIND and Tox21 datasets 9
Table S3. Pharmacophore model coordinates 10

Table S1. Unambiguous Tox21 agonist molecules considered in the study

Molecules screened by one of the 18 pharmacophore models generated are highlighted in green.

PUBCHEM_RESULT_TAG	PUBCHEM_SID	PUBCHEM_CID	PUBCHEM_ACTIVITY_OUTCOME	PUBCHEM_ACTIVITY_SCORE	Activity Summary	Ratio Activity	Ratio Potency (uM)	Ratio Efficacy (%)
**72**	144205785	3033968	Active	56	active agonist	active agonist	0.000648625	44.7551
**124**	144206952	10631	Active	75	active agonist	active agonist	0.000785827	119.711
**156**	144209642	980	Active	80	active agonist	active agonist	0.001	143.371
**220**	144212910	9878	Active	68	active agonist	active agonist	0.001	92.3037
**227**	144213960	13109	Active	55	active agonist	active agonist	0.001	41.2919
**195**	144211847	6238	Active	63	active agonist	active agonist	0.00128805	75.9148
**138**	144207887	6013	Active	88	active agonist	active agonist	0.0014244	179.06
**145**	144208410	9904	Active	71	active agonist	active agonist	0.00165644	111.574
**218**	144212744	6230	Active	58	active agonist	active agonist	0.00223835	58.7889
**194**	144211772	9782	Active	64	active agonist	active agonist	0.00227281	82.5659
**14**	144204071	443935	Active	61	active agonist	active agonist	0.00256454	71.6766
**60**	144205410	68947	Active	78	active agonist	active agonist	0.00258222	145.444
**81**	144206014	27812	Active	59	active agonist	active agonist	0.00268325	63.4127
**77**	144205996	66359	Active	71	active agonist	active agonist	0.00301065	117.705
**137**	144207737	25015	Active	67	active agonist	active agonist	0.00325022	102.331
**142**	144208185	10635	Active	69	active agonist	active agonist	0.00329688	110.444
**7**	144203910	6231	Active	60	active agonist	active agonist	0.0036225	71.5271
**73**	144205803	444008	Active	64	active agonist	active agonist	0.00379019	88.2847
**94**	144206325	14708	Active	63	active agonist	active agonist	0.00393847	84.2886
**24**	144204418	440707	Active	67	active agonist	active agonist	0.00393847	102.973

**133**	144207161	5284587	Active	59	active agonist	active agonist	0.00495824	67.7005
**228**	144214019	6010	Active	75	active agonist	active agonist	0.00504666	141.776
**116**	144206781	3034658	Active	75	active agonist	active agonist	0.00578088	145.211
**211**	144212605	16533	Active	63	active agonist	active agonist	0.00640565	87.5693
**55**	144205191	9677	Active	71	active agonist	active agonist	0.00700369	127.15
**87**	144206191	6917715	Active	58	active agonist	active agonist	0.0072777	67.3898
**15**	144204077	247839	Active	71	active agonist	active agonist	0.00842704	130.459
**104**	144206667	14743	Active	84	active agonist	active agonist	0.00870756	197.859
**209**	144212587	5995	Active	82	active agonist	active agonist	0.00977052	188.113
**139**	144208013	5754	Active	64	active agonist	active agonist	0.0102642	95.6772
**118**	144206893	229455	Active	77	active agonist	active agonist	0.01028	163.422
**90**	144206222	231084	Active	78	active agonist	active agonist	0.0106822	169.661
**69**	144205669	20469	Active	63	active agonist	active agonist	0.0119856	96.191
**26**	144204426	71414	Active	61	active agonist	active agonist	0.0145209	85.9625
**10**	144203988	224246	Active	67	active agonist	active agonist	0.0149856	117.168
**200**	144212257	28417	Active	57	active agonist	active agonist	0.0160903	67.3858
**120**	144206895	31378	Active	70	active agonist	active agonist	0.0162927	133.941
**92**	144206313	68952	Active	71	active agonist	active agonist	0.0162927	137.702
**206**	144212471	5833	Active	59	active agonist	active agonist	0.0184045	76.5593
**203**	144212335	6741	Active	62	active agonist	active agonist	0.0194938	96.7905
**196**	144211864	439501	Active	52	active agonist	active agonist	0.022421	40.2118
**98**	144206491	636398	Active	64	active agonist	active agonist	0.0230141	106.309
**106**	144206694	5876	Active	67	active agonist	active agonist	0.0239145	122.796
**208**	144212586	441207	Active	53	active agonist	active agonist	0.0247039	47.8229
**128**	144207031	5745	Active	58	active agonist	active agonist	0.0253315	75.5809
**61**	144205416	155143	Active	71	active agonist	active agonist	0.0258222	146.503
**59**	144205275	5877	Active	58	active agonist	active agonist	0.0278822	76.7288

**222**	144213304	5755	Active	62	active agonist	active agonist	0.029736	101.938
**27**	144204427	5952	Active	74	active agonist	active agonist	0.0301065	165.61
**70**	144205769	68873	Active	73	active agonist	active agonist	0.0301065	163.515
**84**	144206153	248271	Active	57	active agonist	active agonist	0.0325083	72.4677
**113**	144206719	21700	Active	64	active agonist	active agonist	0.0337801	110.851
**86**	144206181	443936	Active	60	active agonist	active agonist	0.0351016	87.6863
**100**	144206581	71470	Active	62	active agonist	active agonist	0.0379019	101.342
**102**	144206625	5702068	Active	62	active agonist	active agonist	0.0379019	104.942
**13**	144204069	16490	Active	62	active agonist	active agonist	0.0391149	101.479
**132**	144207117	63049	Active	62	active agonist	active agonist	0.0425266	103.592
**46**	144204950	26133	Active	59	active agonist	active agonist	0.0495824	86.7531
**126**	144206973	65359	Active	61	active agonist	active agonist	0.0578088	99.9483
**129**	144207048	16158	Active	61	active agonist	active agonist	0.0648625	100.613
**96**	144206477	9270	Active	54	active agonist	active agonist	0.0756242	59.7639
**193**	144211757	222786	Active	63	active agonist	active agonist	0.0776062	121.639
**135**	144207269	5281034	Active	57	active agonist	active agonist	0.0814027	83.6766
**140**	144208052	6446	Active	67	active agonist	active agonist	0.0843204	144.894
**95**	144206364	6540478	Active	57	active agonist	active agonist	0.0848517	84.0618
**201**	144212286	5284486	Active	53	active agonist	active agonist	0.0870756	57.1597
**215**	144212719	6166	Active	71	active agonist	active agonist	0.0904822	172.962
**19**	144204238	6714002	Active	51	active agonist	active agonist	0.0909931	42.8088
**23**	144204404	5282494	Active	58	active agonist	active agonist	0.0952052	85.9906
**184**	144210627	5994	Active	70	active agonist	active agonist	0.0977004	166.694
**35**	144204576	2724385	Active	49	active agonist	active agonist	0.1028	28.8159
**97**	144206482	91670	Active	68	active agonist	active agonist	0.124545	162.83
**182**	144210516	5865	Active	59	active agonist	active agonist	0.13281	102.808
**89**	144206221	111332	Active	58	active agonist	active agonist	0.15089	97.138

**17**	144204183	5282493	Active	60	active agonist	active agonist	0.15089	107.657
**53**	144205152	11876263	Active	62	active agonist	active agonist	0.162927	126.768
**21**	144204277	636374	Active	55	active agonist	active agonist	0.188658	75.499
**44**	144204881	5834	Active	58	active agonist	active agonist	0.197391	100.959
**99**	144206578	229295	Active	53	active agonist	active agonist	0.197391	61.2609
**105**	144206681	60196346	Active	56	active agonist	active agonist	0.213138	87.5614
**56**	144205200	6300	Active	66	active agonist	active agonist	0.239145	166.106
**78**	144206006	40973	Active	48	active agonist	active agonist	0.239145	28.7062
**216**	144212723	656583	Active	50	active agonist	active agonist	0.26499	42.7332
**226**	144213947	5753	Active	61	active agonist	active agonist	0.291709	125.972
**71**	144205772	444025	Active	49	active agonist	active agonist	0.364749	38.888
**186**	144210896	6128	Active	67	active agonist	active agonist	0.388952	190.293
**210**	144212603	11273	Active	61	active agonist	active agonist	0.419981	135.259
**88**	144206200	9568628	Active	59	active agonist	active agonist	0.425266	122.034
**136**	144207339	6279	Active	55	active agonist	active agonist	0.436145	89.2318
**9**	144203980	9051	Active	54	active agonist	active agonist	0.438876	80.8287
**125**	144206954	656804	Active	56	active agonist	active agonist	0.441903	100.412
**213**	144212641	5284557	Active	60	active agonist	active agonist	0.528725	133.415
**144**	144208409	68289	Active	56	active agonist	active agonist	0.577411	103.833
**49**	144205052	60198	Active	68	active agonist	active agonist	0.600704	215.297
**198**	144212039	108956	Active	48	active agonist	active agonist	0.609064	30.0663
**48**	144205004	13789	Active	59	active agonist	active agonist	0.700369	135.682
**121**	144206907	10041070	Active	59	active agonist	active agonist	0.916208	145.051
**31**	144204447	30323	Active	59	active agonist	active agonist	0.952052	145.996
**80**	144206008	6918178	Active	52	active agonist	active agonist	0.952052	74.1174
**224**	144213522	26041	Active	52	active agonist	active agonist	0.989298	71.7675

Table S2. PDB structures used for docking

PDB	Protein	Exp	Resolution	Chain	Profile	Smiles
1T5Z	AR	X-ray	2.30	A	agonist	C[C@]12CC[C@H]3[C@@H](CC[C@H]4CC(=O)CC[C@]34C)[C@@H]1CC[C@@H]2O
1T63	AR	X-ray	2.07	A	agonist	C[C@]12CC[C@H]3[C@@H](CC[C@H]4CC(=O)CC[C@]34C)[C@@H]1CC[C@@H]2O
1T65	AR	X-ray	1.66	A	agonist	C[C@]12CC[C@H]3[C@@H](CC[C@H]4CC(=O)CC[C@]34C)[C@@H]1CC[C@@H]2O
1XJ7	AR	X-ray	2.70	A	agonist	C[C@]12CC[C@H]3[C@@H](CC[C@H]4CC(=O)CC[C@]34C)[C@@H]1CC[C@@H]2O
1XOW	AR	X-ray	1.80	A	agonist	C[C@]1(O)CC[C@H]2[C@@H]3CCC4=CC(=O)CCC4=C3C=C[C@]12C
1XQ3	AR	X-ray	2.25	A	agonist	C[C@]1(O)CC[C@H]2[C@@H]3CCC4=CC(=O)CCC4=C3C=C[C@]12C
2AM9	AR	X-ray	1.64	A	agonist	C[C@]12CC[C@H]3[C@@H](CCC4=CC(=O)CC[C@]34C)[C@@H]1CC[C@@H]2O
2AMA	AR	X-ray	1.90	A	agonist	C[C@]12CC[C@H]3[C@@H](CC[C@H]4CC(=O)CC[C@]34C)[C@@H]1CC[C@@H]2O
2AMB	AR	X-ray	1.75	A	agonist	CC[C@]1(O)CC[C@H]2[C@@H]3CCC4=CC(=O)CCC4=C3C=C[C@]12CC
2AO6	AR	X-ray	1.89	A	agonist	C[C@]1(O)CC[C@H]2[C@@H]3CCC4=CC(=O)CCC4=C3C=C[C@]12C
2AX9	AR	X-ray	1.65	A	agonist	C[C@](O)(CBr)C(=O)Nc1ccc(c(c1)C(F)(F)F)[N+]([O-])=O
2AXA	AR	X-ray	1.80	A	agonist	C[C@](O)(COc1ccc(F)cc1)C(=O)Nc1ccc(c(c1)C(F)(F)F)[N+]([O-])=O
2PIO	AR	X-ray	2.03	A	agonist	C[C@]12CC[C@H]3[C@@H](CC[C@H]4CC(=O)CC[C@]34C)[C@@H]1CC[C@@H]2O
2PIP	AR	X-ray	1.80	L	agonist	C[C@]12CC[C@H]3[C@@H](CC[C@H]4CC(=O)CC[C@]34C)[C@@H]1CC[C@@H]2O
2PIQ	AR	X-ray	2.40	A	agonist	C[C@]12CC[C@H]3[C@@H](CC[C@H]4CC(=O)CC[C@]34C)[C@@H]1CC[C@@H]2O
2PIR	AR	X-ray	2.10	A	agonist	C[C@]12CC[C@H]3[C@@H](CC[C@H]4CC(=O)CC[C@]34C)[C@@H]1CC[C@@H]2O
2PIT	AR	X-ray	1.76	A	agonist	C[C@]12CC[C@H]3[C@@H](CC[C@H]4CC(=O)CC[C@]34C)[C@@H]1CC[C@@H]2O
2PIU	AR	X-ray	2.12	A	agonist	C[C@]12CC[C@H]3[C@@H](CC[C@H]4CC(=O)CC[C@]34C)[C@@H]1CC[C@@H]2O
2PIV	AR	X-ray	1.95	A	agonist	C[C@]12CC[C@H]3[C@@H](CC[C@H]4CC(=O)CC[C@]34C)[C@@H]1CC[C@@H]2O
2PIW	AR	X-ray	2.58	A	agonist	C[C@]12CC[C@H]3[C@@H](CC[C@H]4CC(=O)CC[C@]34C)[C@@H]1CC[C@@H]2O
2PIX	AR	X-ray	2.40	A	agonist	C[C@]12CC[C@H]3[C@@H](CC[C@H]4CC(=O)CC[C@]34C)[C@@H]1CC[C@@H]2O
2PKL	AR	X-ray	2.49	A	agonist	C[C@]12CC[C@H]3[C@@H](CC[C@H]4CC(=O)CC[C@]34C)[C@@H]1CC[C@@H]2O
2PNU	AR	X-ray	1.65	A	agonist	C[C@]12CCC(=O)C[C@@H]1CC[C@H]1[C@@H]3CC[C@H](O)[C@@]3(CCOCc3cc(F)cc(F)c3)CC[C@H]21
2Q7I	AR	X-ray	1.87	A	agonist	C[C@]12CC[C@H]3[C@@H](CCC4=CC(=O)CC[C@]34C)[C@@H]1CC[C@@H]2O

**2Q7J**	AR	X-ray	1.90	A	agonist	C[C@]12CC[C@H]3[C@@H](CCC4=CC(=O)CC[C@]34C)[C@@H]1CC[C@@H]2O
**2Z4J**	AR	X-ray	2.60	A	agonist	C[C@]12CC[C@H]3[C@@H](CC[C@H]4CC(=O)CC[C@]34C)[C@@H]1CC[C@@H]2O
**3L3X**	AR	X-ray	1.55	A	agonist	C[C@]12CC[C@H]3[C@@H](CC[C@H]4CC(=O)CC[C@]34C)[C@@H]1CC[C@@H]2O
**3L3Z**	AR	X-ray	2.00	A	agonist	C[C@]12CC[C@H]3[C@@H](CC[C@H]4CC(=O)CC[C@]34C)[C@@H]1CC[C@@H]2O
**5JJM**	AR	X-ray	2.15	A, B, C, D	agonist	C[C@]12CC[C@H]3[C@@H](CC[C@H]4CC(=O)CC[C@]34C)[C@@H]1CC[C@@H]2O

Figure S1. Structural Network Analysis

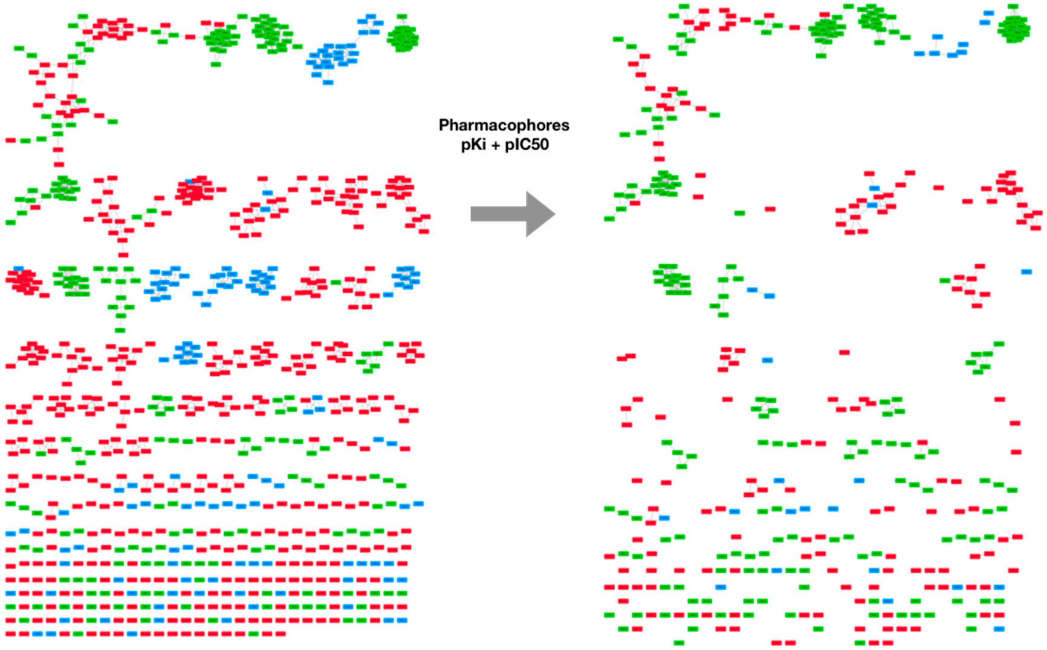

Molecular networks of molecules sharing structural similarity. Colored rounded rectangles represent molecules (green: agonist, red: antagonist, blue: non binder). Each edge connects two molecules with a Tc >= 0.90. The right panel highlights the compounds that have been screened with the ensemble of pKi and pIC50 phermacophore models.

Figure S2. Chemical Space Analysis (PCA) of the NR-DBIND and Tox21 datasets

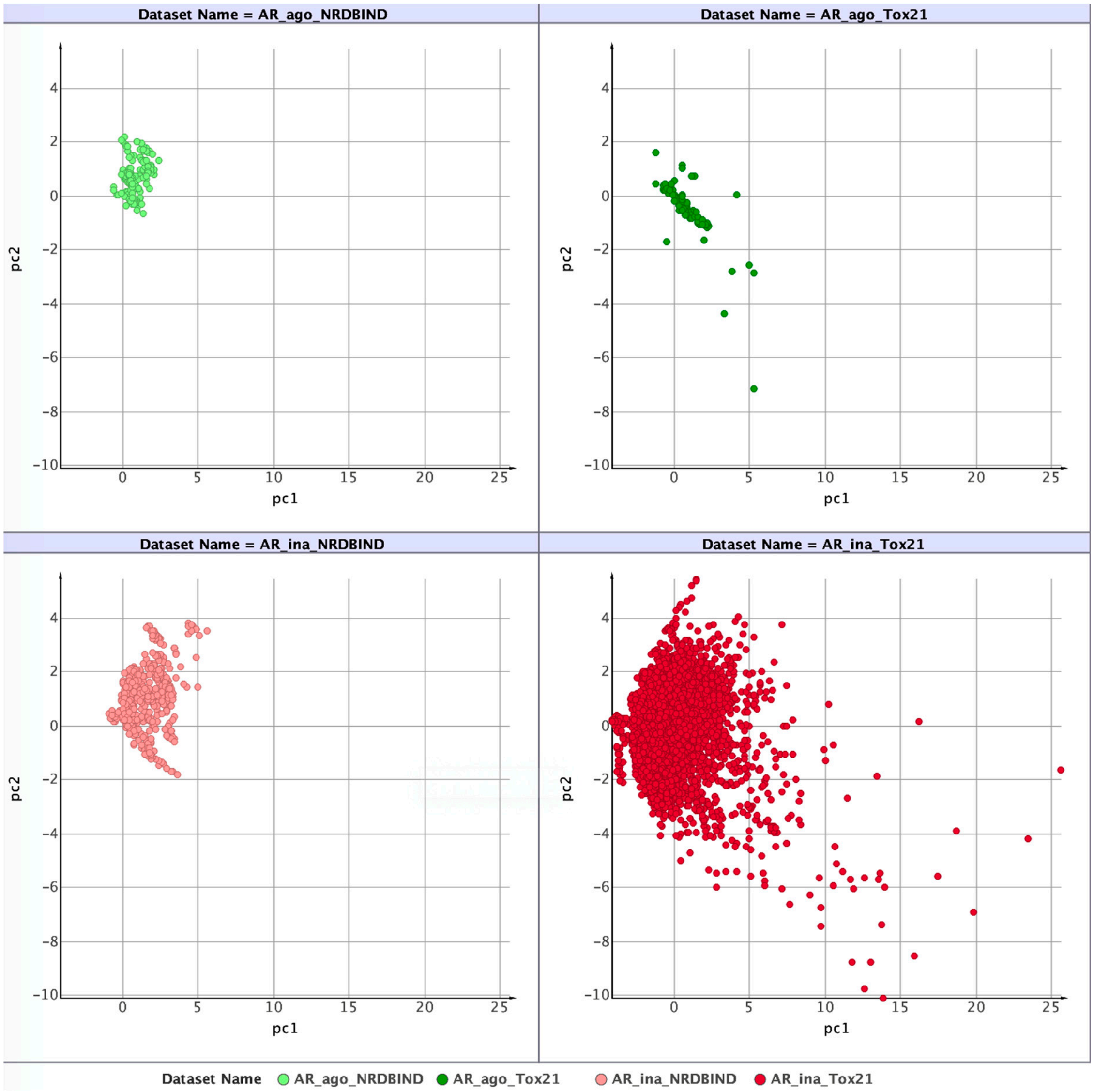

Principal component analysis of the NR-DBIND (left) and Tox21 (right) datasets used for the study. Classical descriptors (molecular weight, cLogP, number of H-bond donor, number of H-bond acceptor, total surface area, total polar surface area, MolFlex, MolComplex, number of rotatable bonds, number of aromatic rings, number of aromatic atoms) were computed with DataWarrior for each molecules, and used to perform the principal component analysis with DataWarrior. The agonist (green) and non-agonist (red) compounds are plotted on the 2 first PCA axes covering respectively 41% and 21% of the variance. Agonist and non-agonist molecules of the NR-DBIND are overlapping. Agonist compounds from the Tox21 do not significantly cover the chemical space of agonist compounds from the NR-DBIND.

Table S3. Pharmacophore model coordinates

AR_agonist_pIC50_1.xyz
type x y z tolerance weight additional_flag
H -2.9198 -2.233 1.7059 1.9499999 1.0
H -1.548751 0.485183 2.418983 1.65 1.0
H 0.026497 -2.481257 -1.100548 1.8 1.0
H -5.340819 1.037824 -4.330588 1.5 1.0 optional
AR -1.3839295 -0.03724755 2.523065 1.3499999 1.0 optional
HBA -0.7136 -0.302 -3.2851 1.0 1.0 optional
HBA -4.6358213 -0.08600117 5.9669733 1.2 1.0
HBA -2.9672925 0.33049953 4.7568355 1.5 0.6 optional
exclusion -5.293088 -2.0412688 -0.61673087 0.70000005 1.0
exclusion -3.3181 2.4367 1.9151 0.5500001 1.0
exclusion -3.1410472 2.2917879 -0.39625537 0.5500001 1.0
exclusion -4.4484153 -4.604938 1.77696 0.70000005 1.0
exclusion -6.4796 -0.5423 0.6502 1.0 1.0
exclusion -2.1395998 -5.3094363 0.73701465 1.0 1.0
exclusion -4.627818 -4.460462 -0.685709 1.0 1.0
exclusion -0.998721 -0.6687772 5.442823 0.5500001 1.0
exclusion -5.6358223 2.5116034 0.19709301 1.5 1.0
exclusion -2.7919 -5.9426 2.8556 1.0 1.0
exclusion 0.5452 0.014 4.9244 1.0 1.0
exclusion 0.3641777 -3.4883966 4.697093 1.5 1.0
exclusion -6.46607 -2.786647 -1.8006656 0.70000005 1.0
exclusion -3.4191852 -2.6506124 -3.9097943 1.0 1.0
exclusion -5.7515216 -1.5225985 5.3207 0.3 1.0
exclusion 1.4587915 -4.8365192 -0.11959292 0.70000005 1.0
exclusion -7.945017 -1.5594493 3.594639 1.0 1.0
exclusion -0.1099 3.287 -1.9868 1.0 1.0
exclusion -4.6001 -3.1043 6.4186 1.0 1.0
exclusion -1.3476417 2.5284102 5.7501097 1.0 1.0
exclusion -2.2901 4.2627 4.1325 1.0 1.0
exclusion 2.6846204 -1.6315789 -2.5315604 0.70000005 1.0
exclusion -7.07831 -3.0160356 5.300307 1.0 1.0
exclusion 1.7265 3.5619 0.194 0.70000005 1.0
exclusion -2.6358223 4.0116034 -2.802907 1.5 1.0
exclusion -3.8742 3.3511 5.5871 0.85 1.0
exclusion 0.8228 -2.8134 -4.4829 1.0 1.0
exclusion 3.8653 -2.7423 2.7422 1.0 1.0
exclusion 3.8862178 -0.63308275 -1.0083754 1.0 1.0
exclusion 3.4406161 -1.4336029 4.3236413 1.0 1.0
exclusion -4.1358223 -7.9883966 1.697093 1.5 1.0
exclusion 2.705726 -4.5239234 -2.7431297 1.0 1.0
exclusion -1.1358223 -7.9883966 -1.302907 1.5 1.0
exclusion -4.1358223 5.5116034 3.197093 1.5 1.0
exclusion -2.6358223 -6.4883966 6.197093 1.5 1.0
exclusion 0.3641777 5.5116034 0.19709301 1.5 1.0
exclusion 0.3641777 -7.9883966 3.197093 1.5 1.0
exclusion -10.135822 -0.48839664 1.697093 1.5 1.0
exclusion 1.8641777 4.0116034 4.697093 1.5 1.0
exclusion 3.3641777 2.5116034 -2.802907 1.5 1.0
exclusion -7.1358223 -4.9883966 -4.302907 1.5 1.0
exclusion 1.8641777 1.0116034 7.697093 1.5 1.0
exclusion -1.1358223 -1.9883966 9.197093 1.5 1.0
exclusion -10.135822 -3.4883966 -1.302907 1.5 1.0
exclusion -8.635822 4.0116034 3.197093 1.5 1.0
exclusion 0.3641777 2.5116034 -5.802907 1.5 1.0
exclusion -2.6358223 4.0116034 7.697093 1.5 1.0
exclusion -7.1358223 -7.9883966 -1.302907 1.5 1.0
exclusion -10.135822 -3.4883966 4.697093 1.5 1.0
exclusion -4.1358223 -7.9883966 -4.302907 1.5 1.0
exclusion -7.1358223 -4.9883966 7.697093 1.5 1.0
exclusion -7.1358223 -7.9883966 4.697093 1.5 1.0
exclusion -5.6358223 -1.9883966 -7.302907 1.5 1.0
exclusion 4.8641777 4.0116034 1.697093 1.5 1.0
exclusion 3.3641777 -1.9883966 -5.802907 1.5 1.0
exclusion -2.6358223 -4.9883966 -7.302907 1.5 1.0
exclusion 3.3641777 -7.9883966 -1.302907 1.5 1.0
exclusion -10.135822 -6.4883966 1.697093 1.5 1.0
exclusion -10.135822 1.0116034 6.197093 1.5 1.0
exclusion 1.8641777 -6.4883966 -5.802907 1.5 1.0
exclusion 6.3641777 -0.48839664 -2.802907 1.5 1.0
exclusion 4.8641777 -6.4883966 4.697093 1.5 1.0
exclusion -4.1358223 1.0116034 10.697093 1.5 1.0
exclusion 7.2814 -1.6627 3.3877 1.0 1.0
exclusion -7.773383 4.8079166 -5.028417 1.15 1.0
exclusion -2.6358223 1.0116034 -8.802907 1.5 1.0
exclusion -5.6437 3.9592 -7.0959 1.0 1.0
exclusion 6.3641777 -1.9883966 6.197093 1.5 1.0
exclusion 0.3641777 -1.9883966 -8.802907 1.5 1.0
exclusion 6.3641777 -4.9883966 -2.802907 1.5 1.0
exclusion -4.1358223 -4.9883966 10.697093 1.5 1.0
exclusion -7.1358223 5.5116034 7.697093 1.5 1.0
exclusion 7.8641777 1.0116034 1.697093 1.5 1.0
exclusion -8.1142845 0.6358953 -8.090595 1.0 1.0
exclusion 7.8641777 -4.9883966 1.697093 1.5 1.0
exclusion -10.135822 -1.9883966 9.197093 1.5 1.0
exclusion -8.635822 2.5116034 10.697093 1.5 1.0
exclusion -7.1358223 -1.9883966 12.197093 1.5 1.0

AR_agonist_pIC50_2.xyz
type x y z tolerance weight additional_flag
H -4.878049 -1.9267721 1.5680003 1.5 1.0
H 3.3174267 -2.1993847 2.1833172 1.5 1.0
HBA 1.4906029 -1.342783 1.9695095 1.5 0.6 optional
HBA 1.3689609 -2.528245 0.25184643 1.5 1.0
HBA -0.58239555 -1.8389704 -1.7128992 1.8 1.0
HBA 3.2769208 -1.4075683 0.72404313 1.5 0.8 optional
HBA -4.481324 -0.027195176 5.8645244 1.5 1.0
exclusion 0.12195206 -3.528245 5.865656 1.5 1.0
exclusion -2.878048 -8.028245 1.3656559 1.5 1.0
exclusion 0.12195206 -8.028245 4.365656 1.5 1.0
exclusion -7.378048 2.471755 2.865656 1.5 1.0
exclusion 1.621952 -8.028245 -0.1343441 1.5 1.0
exclusion 0.12195206 5.471755 1.3656559 1.5 1.0
exclusion -2.878048 -6.528245 7.365656 1.5 1.0
exclusion -4.378048 5.471755 2.865656 1.5 1.0
exclusion 1.621952 3.971755 5.865656 1.5 1.0
exclusion -7.378048 2.471755 -1.6343441 1.5 1.0
exclusion 4.621952 0.971755 5.865656 1.5 1.0
exclusion 1.621952 -0.528245 8.865656 1.5 1.0
exclusion -4.378048 2.471755 -4.634344 1.5 1.0
exclusion -5.878048 -8.028245 4.365656 1.5 1.0
exclusion -2.878048 3.971755 7.365656 1.5 1.0
exclusion -7.378048 -5.028245 -3.134344 1.5 1.0
exclusion -4.378048 -8.028245 -3.134344 1.5 1.0
exclusion 3.121952 -5.028245 -4.634344 1.5 1.0
exclusion -7.378048 -0.528245 -4.634344 1.5 1.0
exclusion -1.378048 5.471755 -3.134344 1.5 1.0
exclusion 4.621952 3.971755 -0.1343441 1.5 1.0
exclusion -7.378048 -8.028245 -0.1343441 1.5 1.0
exclusion -7.378048 -5.028245 7.365656 1.5 1.0
exclusion 3.121952 -6.528245 7.365656 1.5 1.0
exclusion -4.378048 -5.028245 -6.134344 1.5 1.0
exclusion -10.378048 -0.528245 2.865656 1.5 1.0
exclusion 3.121952 2.471755 -4.634344 1.5 1.0
exclusion -1.378048 -3.528245 10.365656 1.5 1.0
exclusion 4.621952 -8.028245 2.865656 1.5 1.0
exclusion 0.12195206 -8.028245 -4.634344 1.5 1.0
exclusion -2.878048 0.971755 10.365656 1.5 1.0
exclusion -10.378048 -0.528245 -1.6343441 1.5 1.0
exclusion 7.621952 -2.028245 -0.1343441 1.5 1.0
exclusion -2.878048 -0.528245 -7.634344 1.5 1.0
exclusion -10.378048 -5.028245 -0.1343441 1.5 1.0
exclusion -5.878048 -2.028245 10.365656 1.5 1.0
exclusion 6.121952 -6.528245 -1.6343441 1.5 1.0
exclusion -10.378048 -5.028245 4.365656 1.5 1.0

exclusion 6.121952 -3.528245 7.365656 1.5 1.0
exclusion 7.621952 -5.028245 2.865656 1.5 1.0
exclusion 6.121952 -2.028245 -4.634344 1.5 1.0
exclusion 0.12195206 -5.028245 -7.634344 1.5 1.0
exclusion -7.378048 5.471755 5.865656 1.5 1.0
exclusion 7.621952 2.471755 2.865656 1.5 1.0
exclusion 0.12195206 2.471755 -7.634344 1.5 1.0
exclusion 3.121952 -2.028245 -7.634344 1.5 1.0
exclusion -10.378048 -2.028245 7.365656 1.5 1.0
exclusion -10.378048 2.471755 5.865656 1.5 1.0
exclusion 7.621952 2.471755 -3.134344 1.5 1.0
exclusion -5.878048 3.971755 10.365656 1.5 1.0
exclusion -8.878048 0.971755 10.365656 1.5 1.0

AR_agonist_pIC50_3.xyz
type x y z tolerance weight additional_flag
H -0.105062515 -1.179263 0.1378745 1.5 1.0
H -4.878049 -1.9267721 1.5680003 1.5 1.0
H -1.3866 0.6644 -1.7227 1.5 1.0 optional
H 3.3174267 -2.1993847 2.1833172 1.5 1.0 optional
HBA 1.4906029 -1.342783 1.9695095 1.5 0.6 optional
HBA 1.3689609 -2.528245 0.25184643 1.5 1.0 optional
HBA 0.0746 -2.4358 -2.1944 1.3 1.0
HBA 3.2769208 -1.4075683 0.72404313 1.5 0.8 optional
HBA -4.481324 -0.027195176 5.8645244 1.5 1.0
HBD 0.0746 -2.4358 -2.1944 1.3 1.0
exclusion 1.0568539 1.9167469 0.9933936 1.0 1.0
exclusion -0.2763 2.6214 -0.1839 0.85 1.0
exclusion -4.3077 2.7755 -0.6619 1.0 1.0
exclusion 0.12195206 -3.528245 5.865656 1.5 1.0
exclusion -6.4468 -4.2524 3.7042 0.70000005 1.0
exclusion -7.8602 -3.1071 3.032 1.0 1.0
exclusion -7.7428 -0.8222 4.0929 0.40000007 1.0
exclusion -2.878048 -8.028245 1.3656559 1.5 1.0
exclusion -7.378048 2.471755 2.865656 1.5 1.0
exclusion 2.9774487 -0.8933885 -3.9466786 1.0 1.0
exclusion -0.42314953 0.7521222 -5.308382 1.0 1.0
exclusion -7.6394 0.8035 4.5971 0.70000005 1.0
exclusion 0.12195206 5.471755 1.3656559 1.5 1.0
exclusion 1.3474331 -0.95326644 -5.4101567 0.70000005 1.0
exclusion -7.378048 2.471755 -1.6343441 1.5 1.0
exclusion 0.12195206 -8.028245 4.365656 1.5 1.0
exclusion 1.621952 -8.028245 -0.1343441 1.5 1.0
exclusion -4.378048 5.471755 2.865656 1.5 1.0
exclusion -4.378048 2.471755 -4.634344 1.5 1.0
exclusion 1.621952 3.971755 5.865656 1.5 1.0
exclusion -2.878048 -6.528245 7.365656 1.5 1.0
exclusion -7.378048 -5.028245 -3.134344 1.5 1.0
exclusion 4.621952 0.971755 5.865656 1.5 1.0
exclusion -2.878048 3.971755 7.365656 1.5 1.0
exclusion -7.378048 -0.528245 -4.634344 1.5 1.0
exclusion -1.378048 5.471755 -3.134344 1.5 1.0
exclusion 1.621952 -0.528245 8.865656 1.5 1.0
exclusion -5.878048 -8.028245 4.365656 1.5 1.0
exclusion -4.378048 -8.028245 -3.134344 1.5 1.0
exclusion 3.121952 -5.028245 -4.634344 1.5 1.0
exclusion 4.621952 3.971755 -0.1343441 1.5 1.0
exclusion 3.121952 2.471755 -4.634344 1.5 1.0
exclusion -4.378048 -5.028245 -6.134344 1.5 1.0
exclusion -7.378048 -8.028245 -0.1343441 1.5 1.0
exclusion -10.378048 -0.528245 2.865656 1.5 1.0
exclusion -7.378048 -5.028245 7.365656 1.5 1.0
exclusion -2.878048 -0.528245 -7.634344 1.5 1.0
exclusion 0.12195206 -8.028245 -4.634344 1.5 1.0
exclusion -10.378048 -0.528245 -1.6343441 1.5 1.0
exclusion 3.121952 -6.528245 7.365656 1.5 1.0
exclusion 4.621952 -8.028245 2.865656 1.5 1.0
exclusion -1.378048 -3.528245 10.365656 1.5 1.0
exclusion -10.378048 -5.028245 -0.1343441 1.5 1.0
exclusion -2.878048 0.971755 10.365656 1.5 1.0
exclusion 7.621952 -2.028245 -0.1343441 1.5 1.0
exclusion 0.12195206 -5.028245 -7.634344 1.5 1.0
exclusion 6.121952 -6.528245 -1.6343441 1.5 1.0
exclusion -10.378048 -5.028245 4.365656 1.5 1.0
exclusion 6.121952 -2.028245 -4.634344 1.5 1.0
exclusion 0.12195206 2.471755 -7.634344 1.5 1.0
exclusion -5.878048 -2.028245 10.365656 1.5 1.0
exclusion -7.378048 5.471755 5.865656 1.5 1.0
exclusion 6.121952 -3.528245 7.365656 1.5 1.0
exclusion 7.621952 -5.028245 2.865656 1.5 1.0
exclusion 3.121952 -2.028245 -7.634344 1.5 1.0
exclusion 7.621952 2.471755 2.865656 1.5 1.0
exclusion -10.378048 2.471755 5.865656 1.5 1.0
exclusion -10.378048 -2.028245 7.365656 1.5 1.0
exclusion 7.621952 2.471755 -3.134344 1.5 1.0
exclusion -5.878048 3.971755 10.365656 1.5 1.0
exclusion -8.878048 0.971755 10.365656 1.5 1.0

AR_agonist_pIC50_4.xyz
type x y z tolerance weight additional_flag
H -1.1157844 -3.1968117 -1.8414524 1.5 0.9
H -4.878049 -1.9267721 1.5680003 1.65 1.0
H 3.3174267 -2.1993847 2.1833172 1.65 1.0 optional
H 3.982065 -4.637866 1.084596 1.65 1.0 optional
HBA 1.3689609 -2.528245 0.25184643 1.5 1.0 optional
HBA 0.0746 -2.4358 -2.1944 1.5999999 1.0
HBA -4.481324 -0.027195176 5.8645244 1.5 1.0
HBA 4.0726 -5.7427 2.1088 1.4499999 1.0 optional
HBD 0.0746 -2.4358 -2.1944 1.5999999 1.0
exclusion 0.6718311 -5.879855 0.38533354 0.5500001 1.0
exclusion 0.07695001 -5.49255 -1.0778999 0.3 1.0
exclusion 1.0568539 1.9167469 0.9933936 1.0 1.0
exclusion 2.3925571 0.061763123 3.8795493 1.0 1.0
exclusion -0.2763 2.6214 -0.1839 0.85 1.0
exclusion 0.12195206 -3.528245 5.865656 1.5 1.0
exclusion 2.0722547 -4.384985 -2.4863462 0.475 1.0
exclusion -4.3077 2.7755 -0.6619 1.0 1.0
exclusion 0.6475 2.3914 -2.7504 1.0 1.0
exclusion -2.878048 -8.028245 1.3656559 1.5 1.0
exclusion -6.4468 -4.2524 3.7042 0.5500001 1.0
exclusion 4.506343 -2.4397821 4.837199 0.9250001 1.0
exclusion 2.9774487 -0.8933885 -3.9466786 1.0 1.0
exclusion 1.621952 -8.028245 -0.1343441 1.5 1.0
exclusion -6.6974 -2.2391 5.163 0.70000005 1.0
exclusion 0.12195206 -8.028245 4.365656 1.5 1.0
exclusion -7.8602 -3.1071 3.032 1.0 1.0
exclusion -0.42314953 0.7521222 -5.308382 1.0 1.0
exclusion 1.3474331 -0.95326644 -5.4101567 0.70000005 1.0
exclusion -7.7428 -0.8222 4.0929 0.40000007 1.0
exclusion 0.12195206 5.471755 1.3656559 1.5 1.0
exclusion -2.878048 -6.528245 7.365656 1.5 1.0
exclusion 4.621952 0.971755 5.865656 1.5 1.0
exclusion 3.121952 -5.028245 -4.634344 1.5 1.0
exclusion -7.6394 0.8035 4.5971 0.70000005 1.0
exclusion -7.378048 2.471755 2.865656 1.5 1.0
exclusion 1.621952 3.971755 5.865656 1.5 1.0
exclusion -2.1342 -6.4166 -5.3384 1.0 1.0
exclusion 1.621952 -0.528245 8.865656 1.5 1.0
exclusion -4.378048 2.471755 -4.634344 1.5 1.0
exclusion -4.378048 -8.028245 -3.134344 1.2 1.0
exclusion -7.378048 2.471755 -1.6343441 1.5 1.0
exclusion -4.378048 5.471755 2.865656 1.5 1.0
exclusion 4.621952 3.971755 -0.1343441 1.5 1.0
exclusion -5.878048 -8.028245 4.365656 1.5 1.0
exclusion -7.378048 -5.028245 -3.134344 1.5 1.0
exclusion 3.121952 2.471755 -4.634344 1.5 1.0
exclusion 0.12195206 -8.028245 -4.634344 1.5 1.0
exclusion -2.878048 3.971755 7.365656 1.5 1.0
exclusion -1.378048 5.471755 -3.134344 1.5 1.0
exclusion -4.378048 -5.028245 -6.134344 1.2 1.0
exclusion 7.621952 -2.028245 -0.1343441 1.5 1.0
exclusion -7.378048 -0.528245 -4.634344 1.5 1.0

exclusion -7.378048 -8.028245 -0.1343441 1.2 1.0
exclusion 6.121952 -6.528245 -1.6343441 0.60000014 1.0
exclusion -1.378048 -3.528245 10.365656 1.5 1.0
exclusion -2.878048 -0.528245 -7.634344 1.5 1.0
exclusion -7.378048 -5.028245 7.365656 1.5 1.0
exclusion 6.121952 -2.028245 -4.634344 1.5 1.0
exclusion 0.12195206 -5.028245 -7.634344 1.5 1.0
exclusion -10.378048 -0.528245 2.865656 1.5 1.0
exclusion -2.878048 0.971755 10.365656 1.5 1.0
exclusion 7.621952 2.471755 2.865656 1.5 1.0
exclusion 3.121952 -2.028245 -7.634344 1.5 1.0
exclusion -10.378048 -0.528245 -1.6343441 1.5 1.0
exclusion -10.378048 -5.028245 -0.1343441 1.5 1.0
exclusion 0.12195206 2.471755 -7.634344 1.5 1.0
exclusion -5.878048 -2.028245 10.365656 1.5 1.0
exclusion -10.378048 -5.028245 4.365656 1.5 1.0
exclusion 7.621952 2.471755 -3.134344 1.5 1.0
exclusion -7.378048 5.471755 5.865656 1.5 1.0
exclusion -10.378048 -2.028245 7.365656 1.5 1.0
exclusion -10.378048 2.471755 5.865656 1.5 1.0
exclusion -5.878048 3.971755 10.365656 1.5 1.0
exclusion 8.944 -5.7368 6.6717 1.0 1.0
exclusion 5.8225 -11.667 3.364 1.4499999 1.0
exclusion -8.878048 0.971755 10.365656 1.5 1.0
exclusion 8.6164 -9.6329 5.2445 1.0 1.0
exclusion 11.4354 -6.9268 3.2255 1.4499999 1.0

AR_agonist_pIC50_5.xyz
type x y z tolerance weight additional_flag
H -2.4656188 0.21610793 3.6876354 1.5 1.0
H 2.8281 -0.2638 0.6191 1.5 1.0
AR -2.116787 0.025461506 2.5768085 0.8999999 1.0 optional
HBA -5.090538 -2.683052 1.8703 1.5 1.0
HBA -5.090538 -2.683052 1.8703 1.5 1.0
HBA 1.5376356 0.7359357 -1.6168168 1.55 1.0
HBA 2.51505 3.2198 -0.5912 1.0 1.0
HBD -3.8525 -2.3115 1.0883 1.0 1.0 optional
HBD 2.4431 1.143 -1.2819 1.15 1.0
exclusion -3.5980172 2.7061825 -0.13935457 0.85 1.0
exclusion -4.590538 0.81694794 -1.0984335 1.5 1.0
exclusion -5.163 3.5522 -2.3772 1.0 1.0
exclusion 0.5048 -3.5662 -2.7694 1.0 1.0
exclusion -4.956978 -1.6838886 5.7322807 0.5500001 1.0
exclusion 2.909462 -3.683052 1.9015665 1.5 1.0
exclusion -6.090538 5.316948 1.9015665 1.5 1.0
exclusion -4.590538 0.81694794 -5.5984335 1.5 1.0
exclusion -0.090538025 -5.183052 4.9015665 1.5 1.0
exclusion -0.090538025 -6.683052 0.4015665 1.5 1.0
exclusion -3.090538 -5.183052 -4.0984335 1.5 1.0
exclusion -7.590538 2.316948 4.9015665 1.5 1.0
exclusion -9.090538 2.316948 0.4015665 1.5 1.0
exclusion 1.409462 5.316948 4.9015665 1.5 1.0
exclusion -7.590538 -2.183052 -4.0984335 1.5 1.0
exclusion -0.090538025 2.316948 7.9015665 1.5 1.0
exclusion -0.090538025 -2.183052 7.9015665 1.5 1.0
exclusion 1.409462 -5.183052 -4.0984335 1.5 1.0
exclusion 4.409462 2.316948 4.9015665 1.5 1.0
exclusion -4.590538 5.316948 6.4015665 1.5 1.0
exclusion -1.590538 -2.183052 -7.0984335 1.5 1.0
exclusion 5.909462 -0.68305206 1.9015665 1.5 1.0
exclusion -7.590538 5.316948 -2.5984335 1.5 1.0
exclusion 1.409462 0.81694794 -7.0984335 1.5 1.0
exclusion -1.590538 3.816948 -7.0984335 1.5 1.0
exclusion -1.590538 8.316948 3.4015665 1.5 1.0
exclusion -4.590538 -8.183052 0.4015665 1.5 1.0
exclusion 5.909462 3.816948 0.4015665 1.5 1.0
exclusion -10.590538 -0.68305206 3.4015665 1.5 1.0
exclusion 4.409462 -2.183052 6.4015665 1.5 1.0
exclusion -10.590538 -2.183052 -1.0984335 1.5 1.0
exclusion -3.090538 -5.183052 7.9015665 1.5 1.0
exclusion -4.590538 0.81694794 9.4015665 1.5 1.0
exclusion 4.409462 -2.183052 -5.5984335 1.5 1.0
exclusion -7.590538 -6.683052 -2.5984335 1.5 1.0
exclusion -3.090538 -8.183052 4.9015665 1.5 1.0
exclusion 2.909462 8.316948 1.9015665 1.5 1.0
exclusion -4.590538 8.316948 -4.0984335 1.5 1.0
exclusion -4.590538 9.816948 0.4015665 1.5 1.0
exclusion -0.090538025 9.816948 -1.0984335 1.5 1.0
exclusion 7.409462 -0.68305206 -2.5984335 1.5 1.0
exclusion -7.590538 -8.183052 3.4015665 1.5 1.0
exclusion 5.909462 2.316948 -5.5984335 1.5 1.0
exclusion -0.090538025 8.316948 -5.5984335 1.5 1.0
exclusion -7.590538 -5.183052 7.9015665 1.5 1.0
exclusion -10.590538 -5.183052 4.9015665 1.5 1.0
exclusion -10.590538 -6.683052 0.4015665 1.5 1.0
exclusion 4.409462 8.316948 -2.5984335 1.5 1.0

AR_agonist_pIC50_6.xyz
type x y z tolerance weight additional_flag
H -3.4514 1.0771 4.3852 1.5 1.0
H 0.88327503 3.391725 -1.04795 1.65 0.9
H -1.738301 5.508399 0.86265 1.65 0.6999999
HBA -3.2635 -1.6899 0.8343 1.0 1.0 optional
HBA -5.090538 -2.683052 1.8703 1.35 1.0
HBA -2.08235 4.4583 -0.8103 1.0 1.0
HBD -3.9624 -2.519 0.7145 1.0 1.0 optional
HBD 0.49741682 0.80437005 -1.3490461 1.5 0.85
exclusion -4.590538 0.81694794 -1.0984335 1.5 1.0
exclusion 0.908126 0.3742931 1.742094 0.5500001 1.0
exclusion -0.9783 -3.1359 -2.4056 1.0 1.0
exclusion -2.9913013 -2.1621735 -3.3311615 1.0 1.0
exclusion 1.3774 -2.6022 -0.8725 1.0 1.0
exclusion -3.3620198 3.9151378 3.3372664 0.5500001 1.0
exclusion 0.0695 2.7309 4.7602 0.70000005 1.0
exclusion -1.9325 -1.7055 5.8282 0.5500001 1.0
exclusion -3.0908 -5.4261 0.9536 1.0 1.0
exclusion -4.1167383 -2.7405355 5.364767 1.0 1.0
exclusion 1.1681994 -2.1687124 -3.782334 1.0 1.0
exclusion -4.882004 -5.041749 -0.8730904 1.0 1.0
exclusion -6.2331 -2.037 4.4856 1.0 1.0
exclusion -4.6728 -0.4352 6.0685 1.0 1.0
exclusion -7.2728615 -0.43441314 3.83522 1.0 1.0
exclusion -3.243 -5.2722 3.3972 1.0 1.0
exclusion 2.44447 1.9878845 -3.2491229 0.40000007 1.0
exclusion -7.0552 -3.8986 1.5993 0.3 1.0
exclusion 2.3505034 0.17919168 -3.9458914 1.0 1.0
exclusion -8.361377 -1.7143651 0.9787548 1.0 1.0
exclusion -7.1968517 -2.9113777 3.3724964 0.70000005 1.0
exclusion 2.909462 -3.683052 1.9015665 1.5 1.0
exclusion -5.5444 -3.9963 4.3798 1.0 1.0
exclusion -4.590538 0.81694794 -5.5984335 1.5 1.0
exclusion -0.7887 -6.0242 -1.8328 1.0 1.0
exclusion -0.090538025 -6.683052 0.4015665 1.5 1.0
exclusion -3.090538 -5.183052 -4.0984335 1.5 1.0
exclusion -0.090538025 -5.183052 4.9015665 1.5 1.0
exclusion -9.090538 2.316948 0.4015665 1.5 1.0
exclusion -7.590538 2.316948 4.9015665 1.5 1.0
exclusion -7.590538 -2.183052 -4.0984335 1.5 1.0
exclusion 1.409462 5.316948 4.9015665 1.5 1.0
exclusion -0.090538025 2.316948 7.9015665 1.5 1.0
exclusion 1.409462 -5.183052 -4.0984335 1.5 1.0
exclusion -0.090538025 -2.183052 7.9015665 1.5 1.0
exclusion -1.590538 -2.183052 -7.0984335 1.5 1.0
exclusion 2.92025 3.11535 -4.8966 1.0 1.0
exclusion -4.590538 5.316948 6.4015665 1.5 1.0
exclusion 4.409462 2.316948 4.9015665 1.5 1.0
exclusion 5.909462 -0.68305206 1.9015665 1.5 1.0
exclusion 1.409462 0.81694794 -7.0984335 1.5 1.0
exclusion -1.590538 3.816948 -7.0984335 1.5 1.0
exclusion -1.8454 5.8052 6.9856 1.0 1.0
exclusion -4.590538 -8.183052 0.4015665 1.5 1.0
exclusion -1.590538 8.316948 3.4015665 1.5 1.0

exclusion -10.590538 -2.183052 -1.0984335 1.5 1.0
exclusion -10.590538 -0.68305206 3.4015665 1.5 1.0
exclusion 5.909462 3.816948 0.4015665 1.5 1.0
exclusion -3.090538 -5.183052 7.9015665 1.5 1.0
exclusion 4.409462 -2.183052 6.4015665 1.5 1.0
exclusion -4.590538 0.81694794 9.4015665 1.5 1.0
exclusion 1.6654 6.4936 -4.8499 1.0 1.0
exclusion -7.590538 -6.683052 -2.5984335 1.5 1.0
exclusion 4.409462 -2.183052 -5.5984335 1.5 1.0
exclusion -3.090538 -8.183052 4.9015665 1.5 1.0
exclusion 4.352683 5.7230835 -3.2380333 1.0 1.0
exclusion 2.909462 8.316948 1.9015665 1.5 1.0
exclusion -1.4862 6.5713 -6.8669 1.0 1.0
exclusion -0.090538025 9.816948 -1.0984335 1.5 1.0
exclusion 7.409462 -0.68305206 -2.5984335 1.5 1.0
exclusion -0.0642 6.4304 -7.2351 1.0 1.0
exclusion -7.590538 -8.183052 3.4015665 1.5 1.0
exclusion 5.909462 2.316948 -5.5984335 1.5 1.0
exclusion -0.090538025 8.316948 -5.5984335 1.5 1.0
exclusion -7.590538 -5.183052 7.9015665 1.5 1.0
exclusion -10.590538 -6.683052 0.4015665 1.5 1.0
exclusion -10.590538 -5.183052 4.9015665 1.5 1.0
exclusion 4.409462 8.316948 -2.5984335 1.5 1.0

AR_agonist_pIC50_7.xyz
type x y z tolerance weight additional_flag
H -2.256744 -0.8094 0.843231 1.5 1.0
H -2.1117377 0.29971588 2.8835704 1.5 1.0
H 1.14125 1.487675 -0.18205 1.5 1.0 optional
H -1.2755611 4.4307227 -0.011332989 1.5 1.0 optional
AR -2.1329167 -0.6932333 0.79718333 0.9 1.0
AR -2.1329167 -0.6932333 0.79718333 0.9 1.0
AR -2.4868333 0.51523334 2.91135 0.9 1.0
AR -2.4868333 0.51523334 2.91135 0.9 1.0
HBA 1.1029867 2.375317 -1.9777594 1.0 1.0 optional
HBA -5.090538 -2.683052 1.8703 1.0500001 1.0
HBA -5.090538 -2.683052 1.8703 1.0500001 1.0
HBD 1.131 1.4799 -2.4577 1.0 1.0 optional
exclusion -0.3118732 0.38481152 -2.378739 0.40000007 1.0
exclusion 1.354037 -0.55367494 0.64744127 0.3 1.0
exclusion -4.590538 0.81694794 -1.0984335 1.5 1.0
exclusion -2.5508 -1.51265 -2.06995 0.40000007 1.0
exclusion 1.6315 -2.1866 -0.4448 0.70000005 1.0
exclusion 2.7142124 -0.075254664 -0.28047994 0.3 1.0
exclusion -3.9739 4.7248 -1.5559 0.70000005 1.0
exclusion 2.5749 3.2659 2.4316 1.0 1.0
exclusion -2.7073 4.9305 3.6776 1.0 1.0
exclusion 0.3205947 1.1527587 -4.700446 1.0 1.0
exclusion 1.5958765 5.1410017 -0.19463223 0.40000007 1.0
exclusion -4.6587 -2.2232 4.1097 0.3 1.0
exclusion 0.4529 3.9473 -3.8352 0.5500001 1.0
exclusion -2.3359709 6.4811664 1.0484855 0.5500001 1.0
exclusion -0.40982127 5.9751835 2.7868388 1.0 1.0
exclusion -1.2962675 6.741718 0.23532347 0.40000007 1.0
exclusion 4.4618335 1.2397462 -0.55274975 1.0 1.0
exclusion 2.0213642 5.615441 1.0691017 0.5500001 1.0
exclusion 3.3894 -2.9429 -0.9735 0.70000005 1.0
exclusion -2.6712818 3.2471917 -5.1903167 0.70000005 1.0
exclusion 2.909462 -3.683052 1.9015665 1.5 1.0
exclusion 2.3692777 -0.86607534 -4.3872204 1.0 1.0
exclusion -6.090538 5.316948 1.9015665 1.5 1.0
exclusion -4.590538 0.81694794 -5.5984335 1.5 1.0
exclusion -4.5479 -4.0711 4.68125 1.0 1.0
exclusion 4.3122 0.15155001 -3.4522 1.0 1.0
exclusion 4.751 2.0201 -2.4523 1.0 1.0
exclusion 1.409462 5.316948 4.9015665 1.5 1.0
exclusion 2.8857 5.653 -1.9198 1.0 1.0
exclusion -6.494 -0.0094 5.5781 1.0 1.0
exclusion -7.134 -4.203 -0.215 1.0 1.0
exclusion -0.090538025 -5.183052 4.9015665 1.5 1.0
exclusion -0.090538025 -6.683052 0.4015665 1.5 1.0
exclusion -3.090538 -5.183052 -4.0984335 1.5 1.0
exclusion 4.409462 2.316948 4.9015665 1.5 1.0
exclusion -7.590538 2.316948 4.9015665 1.5 1.0
exclusion 5.909462 -0.68305206 1.9015665 1.5 1.0
exclusion -9.090538 2.316948 0.4015665 1.5 1.0
exclusion -0.090538025 2.316948 7.9015665 1.5 1.0
exclusion -5.2368 -2.2749 6.7124 1.0 1.0
exclusion 1.409462 -5.183052 -4.0984335 1.5 1.0
exclusion -1.590538 -2.183052 -7.0984335 1.5 1.0
exclusion -7.590538 -2.183052 -4.0984335 1.5 1.0
exclusion -0.090538025 -2.183052 7.9015665 1.5 1.0
exclusion 5.909462 3.816948 0.4015665 1.5 1.0
exclusion 1.409462 0.81694794 -7.0984335 1.5 1.0
exclusion -4.590538 5.316948 6.4015665 1.5 1.0
exclusion -1.590538 3.816948 -7.0984335 1.5 1.0
exclusion -1.590538 8.316948 3.4015665 1.5 1.0
exclusion -7.590538 5.316948 -2.5984335 1.5 1.0
exclusion 4.409462 -2.183052 6.4015665 1.5 1.0
exclusion 4.409462 -2.183052 -5.5984335 1.5 1.0
exclusion 2.909462 8.316948 1.9015665 1.5 1.0
exclusion -4.590538 -8.183052 0.4015665 1.5 1.0
exclusion -0.090538025 9.816948 -1.0984335 1.5 1.0
exclusion -4.590538 8.316948 -4.0984335 1.5 1.0
exclusion -4.590538 0.81694794 9.4015665 1.5 1.0
exclusion 7.409462 -0.68305206 -2.5984335 1.5 1.0
exclusion -3.090538 -5.183052 7.9015665 1.5 1.0
exclusion -10.590538 -0.68305206 3.4015665 1.5 1.0
exclusion -10.590538 -2.183052 -1.0984335 1.5 1.0
exclusion -4.590538 9.816948 0.4015665 1.5 1.0
exclusion 5.909462 2.316948 -5.5984335 1.5 1.0
exclusion -0.090538025 8.316948 -5.5984335 1.5 1.0
exclusion -7.590538 -6.683052 -2.5984335 1.5 1.0
exclusion -3.090538 -8.183052 4.9015665 1.5 1.0
exclusion 4.409462 8.316948 -2.5984335 1.5 1.0
exclusion -7.590538 -8.183052 3.4015665 1.5 1.0
exclusion -7.590538 -5.183052 7.9015665 1.5 1.0
exclusion -10.590538 -5.183052 4.9015665 1.5 1.0
exclusion -10.590538 -6.683052 0.4015665 1.5 1.0

AR_agonist_pIC50_8.xyz
type x y z tolerance weight additional_flag
H -3.3344 0.936 3.1501 1.5 1.0
AR -1.3079333 -0.40653336 0.68436664 0.9 1.0
AR -3.4715667 -1.311 1.3516166 0.9 1.0
HBA -3.322144 -2.355218 0.249044 1.0 1.0 optional
HBA -5.3487 -3.1598 0.977 1.0 1.0
HBA -5.3487 -3.1598 0.977 1.0 1.0
HBD 0.9492 -0.5764 -0.9835 1.0 1.0
exclusion 0.041781068 1.4559435 -1.6739007 0.3 1.0
exclusion -0.6880145 -0.4216839 -2.91756 0.70000005 1.0
exclusion -4.590538 0.81694794 -1.0984335 1.5 1.0
exclusion -0.6529544 2.152721 -3.176307 0.5500001 1.0
exclusion 1.6709759 -2.5583906 -1.7021456 0.40000007 1.0
exclusion 3.5809526 -1.3432535 0.8431765 0.70000005 1.0
exclusion 1.1612061 3.1900568 -3.9661293 1.0 1.0
exclusion 2.1822643 1.5171382 -4.2002077 0.40000007 1.0
exclusion 0.71207047 -1.4328523 -5.1807055 0.70000005 1.0
exclusion 2.909462 -3.683052 1.9015665 1.5 1.0
exclusion -4.590538 0.81694794 -5.5984335 1.5 1.0
exclusion 3.120185 4.6482716 -1.8489956 0.5500001 1.0
exclusion -3.090538 -5.183052 -4.0984335 1.5 1.0
exclusion -0.090538025 -6.683052 0.4015665 1.5 1.0
exclusion 4.4388714 2.7927876 -2.4253163 0.70000005 1.0
exclusion 5.1919003 0.63335 -1.84235 0.3 1.0
exclusion -6.090538 5.316948 1.9015665 1.5 1.0
exclusion -0.090538025 -5.183052 4.9015665 1.5 1.0
exclusion 1.409462 -5.183052 -4.0984335 1.5 1.0
exclusion -1.590538 -2.183052 -7.0984335 1.5 1.0
exclusion 1.409462 5.316948 4.9015665 1.5 1.0
exclusion -7.590538 -2.183052 -4.0984335 1.5 1.0
exclusion 5.909462 -0.68305206 1.9015665 1.5 1.0

exclusion -9.090538 2.316948 0.4015665 1.5 1.0
exclusion 1.409462 0.81694794 -7.0984335 1.5 1.0
exclusion 4.409462 2.316948 4.9015665 1.5 1.0
exclusion -7.590538 2.316948 4.9015665 1.5 1.0
exclusion -1.590538 3.816948 -7.0984335 1.5 1.0
exclusion -0.090538025 2.316948 7.9015665 1.5 1.0
exclusion -0.090538025 -2.183052 7.9015665 1.5 1.0
exclusion 5.909462 3.816948 0.4015665 1.5 1.0
exclusion -7.590538 5.316948 -2.5984335 1.5 1.0
exclusion 4.409462 -2.183052 -5.5984335 1.5 1.0
exclusion -4.590538 5.316948 6.4015665 1.5 1.0
exclusion -1.590538 8.316948 3.4015665 1.5 1.0
exclusion -4.590538 -8.183052 0.4015665 1.5 1.0
exclusion 4.409462 -2.183052 6.4015665 1.5 1.0
exclusion -10.590538 -2.183052 -1.0984335 1.5 1.0
exclusion 7.409462 -0.68305206 -2.5984335 1.5 1.0
exclusion 2.909462 8.316948 1.9015665 1.5 1.0
exclusion -7.590538 -6.683052 -2.5984335 1.5 1.0
exclusion -3.090538 -5.183052 7.9015665 1.5 1.0
exclusion -10.590538 -0.68305206 3.4015665 1.5 1.0
exclusion -4.590538 8.316948 -4.0984335 1.5 1.0
exclusion 5.909462 2.316948 -5.5984335 1.5 1.0
exclusion -3.090538 -8.183052 4.9015665 1.5 1.0
exclusion -4.590538 0.81694794 9.4015665 1.5 1.0
exclusion -0.090538025 9.816948 -1.0984335 1.5 1.0
exclusion -0.090538025 8.316948 -5.5984335 1.5 1.0
exclusion -4.590538 9.816948 0.4015665 1.5 1.0
exclusion 4.409462 8.316948 -2.5984335 1.5 1.0
exclusion -7.590538 -8.183052 3.4015665 1.5 1.0
exclusion -7.590538 -5.183052 7.9015665 1.5 1.0
exclusion -10.590538 -6.683052 0.4015665 1.5 1.0
exclusion -10.590538 -5.183052 4.9015665 1.5 1.0

AR_agonist_pIC50_9.xyz
type x y z tolerance weight additional_flag
H -2.256744 -0.8094 0.843231 1.5 1.0
H -2.1117377 0.29971588 2.8835704 1.5 1.0
H 1.14125 1.487675 -0.18205 1.5 1.0 optional
H -1.2755611 4.4307227 -0.011332989 1.5 1.0 optional
AR -2.1329167 -0.6932333 0.79718333 0.9 1.0
AR -2.1329167 -0.6932333 0.79718333 0.9 1.0
AR -2.4868333 0.51523334 2.91135 0.9 1.0
AR -2.4868333 0.51523334 2.91135 0.9 1.0
HBA 1.1029867 2.375317 -1.9777594 1.0 1.0 optional
HBA -5.090538 -2.683052 1.8703 1.0500001 1.0
HBA -5.090538 -2.683052 1.8703 1.0500001 1.0
HBD 1.131 1.4799 -2.4577 1.0 1.0 optional
exclusion -4.590538 0.81694794 -1.0984335 1.5 1.0
exclusion -2.5508 -1.51265 -2.06995 0.40000007 1.0
exclusion -1.8326857 1.6538229 -3.7507627 0.40000007 1.0
exclusion 1.6315 -2.1866 -0.4448 0.70000005 1.0
exclusion 2.7142124 -0.075254664 -0.28047994 0.3 1.0
exclusion -3.9739 4.7248 -1.5559 0.40000007 1.0
exclusion 2.5749 3.2659 2.4316 1.0 1.0
exclusion -2.7073 4.9305 3.6776 1.0 1.0
exclusion 0.3205947 1.1527587 -4.700446 1.0 1.0
exclusion 1.5958765 5.1410017 -0.19463223 0.40000007 1.0
exclusion -4.6587 -2.2232 4.1097 0.3 1.0
exclusion 0.4529 3.9473 -3.8352 0.3 1.0
exclusion -2.3359709 6.4811664 1.0484855 0.5500001 1.0
exclusion -0.40982127 5.9751835 2.7868388 1.0 1.0
exclusion -1.2962675 6.741718 0.23532347 0.40000007 1.0
exclusion 2.0213642 5.615441 1.0691017 0.5500001 1.0
exclusion 4.4618335 1.2397462 -0.55274975 1.0 1.0
exclusion -2.6712818 3.2471917 -5.1903167 0.70000005 1.0
exclusion 3.3894 -2.9429 -0.9735 0.70000005 1.0
exclusion 2.3692777 -0.86607534 -4.3872204 1.0 1.0
exclusion 2.909462 -3.683052 1.9015665 1.5 1.0
exclusion -4.577157 -1.787242 5.637041 1.0 1.0
exclusion -6.090538 5.316948 1.9015665 1.5 1.0
exclusion -4.590538 0.81694794 -5.5984335 1.5 1.0
exclusion -4.5479 -4.0711 4.68125 1.0 1.0
exclusion 4.3122 0.15155001 -3.4522 1.0 1.0
exclusion 2.8857 5.653 -1.9198 1.0 1.0
exclusion 4.751 2.0201 -2.4523 1.0 1.0
exclusion 1.409462 5.316948 4.9015665 1.5 1.0
exclusion -6.494 -0.0094 5.5781 1.0 1.0
exclusion -7.134 -4.203 -0.215 1.0 1.0
exclusion -0.090538025 -6.683052 0.4015665 1.5 1.0
exclusion -0.090538025 -5.183052 4.9015665 1.5 1.0
exclusion -3.090538 -5.183052 -4.0984335 1.5 1.0
exclusion 4.409462 2.316948 4.9015665 1.5 1.0
exclusion -7.590538 2.316948 4.9015665 1.5 1.0
exclusion -9.090538 2.316948 0.4015665 1.5 1.0
exclusion 5.909462 -0.68305206 1.9015665 1.5 1.0
exclusion -0.090538025 2.316948 7.9015665 1.5 1.0
exclusion 1.409462 -5.183052 -4.0984335 1.5 1.0
exclusion -7.590538 -2.183052 -4.0984335 1.5 1.0
exclusion -1.590538 -2.183052 -7.0984335 1.5 1.0
exclusion 1.409462 0.81694794 -7.0984335 1.5 1.0
exclusion 5.909462 3.816948 0.4015665 1.5 1.0
exclusion -4.590538 5.316948 6.4015665 1.5 1.0
exclusion -0.090538025 -2.183052 7.9015665 1.5 1.0
exclusion -1.590538 3.816948 -7.0984335 1.5 1.0
exclusion -7.590538 5.316948 -2.5984335 1.5 1.0
exclusion -1.590538 8.316948 3.4015665 1.5 1.0
exclusion 4.409462 -2.183052 6.4015665 1.5 1.0
exclusion 4.409462 -2.183052 -5.5984335 1.5 1.0
exclusion 2.909462 8.316948 1.9015665 1.5 1.0
exclusion -4.590538 -8.183052 0.4015665 1.5 1.0
exclusion -4.590538 8.316948 -4.0984335 1.5 1.0
exclusion -0.090538025 9.816948 -1.0984335 1.5 1.0
exclusion -4.590538 0.81694794 9.4015665 1.5 1.0
exclusion -10.590538 -2.183052 -1.0984335 1.5 1.0
exclusion -10.590538 -0.68305206 3.4015665 1.5 1.0
exclusion 7.409462 -0.68305206 -2.5984335 1.5 1.0
exclusion -4.590538 9.816948 0.4015665 1.5 1.0
exclusion -3.090538 -5.183052 7.9015665 1.5 1.0
exclusion 5.909462 2.316948 -5.5984335 1.5 1.0
exclusion -0.090538025 8.316948 -5.5984335 1.5 1.0
exclusion -7.590538 -6.683052 -2.5984335 1.5 1.0
exclusion -3.090538 -8.183052 4.9015665 1.5 1.0
exclusion 4.409462 8.316948 -2.5984335 1.5 1.0
exclusion -7.590538 -8.183052 3.4015665 1.5 1.0
exclusion -7.590538 -5.183052 7.9015665 1.5 1.0
exclusion -10.590538 -6.683052 0.4015665 1.5 1.0
exclusion -10.590538 -5.183052 4.9015665 1.5 1.0

AR_agonist_pKi_1.xyz
type x y z tolerance weight additional_flag
H -1.7001194 0.27979022 -0.51461804 1.5 1.0
H 1.0111487 -0.6803593 -3.6627116 1.5 1.0
H 2.1654782 1.5565534 -0.24641228 1.35 1.0
H -1.877025 0.056724504 4.4903493 1.5 1.0 optional
AR -1.2215242 0.060515195 -1.8829569 0.8999999 1.0
HBA 1.3659799 -5.551115E-17 1.110223E-16 1.5 1.0 optional
HBA -0.7545196 -1.0558171 1.9792697 1.5 1.0
HBA -2.517291 0.6468353 -5.0968246 1.5 1.0
HBA -2.517291 0.6468353 -5.0968246 1.5 1.0
HBA -4.2668376 0.2590977 -3.9114542 1.5 1.0 optional
HBD -4.3518734 0.27820322 -3.8643675 1.5 0.8 optional
exclusion -3.93295 -2.48975 -0.36124998 0.70000005 1.0
exclusion -3.5485935 1.7570832 1.4885219 1.0 1.0
exclusion -0.117598325 2.6081965 2.4300706 0.5500001 1.0
exclusion -5.0400305 3.5107024 -0.20309341 1.0 1.0
exclusion -3.6702645 -2.0525813 2.893721 1.0 1.0
exclusion -0.8518734 5.4441833 -0.096824646 1.5 1.0
exclusion -3.0468028 -2.9509373 -5.4920006 1.0 1.0
exclusion -6.6333 -0.4417 -2.5402 1.0 1.0
exclusion -1.1009195 2.6516218 4.0896034 0.5500001 1.0
exclusion 4.3660417 2.4529862 -2.2251582 1.0 1.0
exclusion 1.3061 2.9907 3.4449 1.0 1.0

exclusion 2.1481266 3.9441833 -4.5968246 1.5 1.0
exclusion -4.4413 2.2664 3.2445 1.0 1.0
exclusion -0.35395026 -0.16883606 -7.2620335 1.0 1.0
exclusion 2.6739 -1.3559 -6.0772 1.0 1.0
exclusion -3.8518734 5.4441833 -3.0968246 1.5 1.0
exclusion 3.8511586 2.0754263 2.1752548 0.70000005 1.0
exclusion -7.2236166 1.6773332 -2.5415668 1.0 1.0
exclusion 5.1481266 -2.0558167 -1.5968246 1.5 1.0
exclusion -0.8518734 -6.5558167 -1.5968246 1.5 1.0
exclusion -2.5424 2.5023 -7.8933 1.0 1.0
exclusion -6.578859 1.615443 -5.644575 1.0 1.0
exclusion -5.3518734 5.4441833 1.4031754 1.5 1.0
exclusion -3.8518734 -6.5558167 1.4031754 1.5 1.0
exclusion -0.8518734 5.4441833 4.4031754 1.5 1.0
exclusion 1.705 -3.7667 4.9132 1.0 1.0
exclusion -6.8518734 -5.0558167 -1.5968246 1.5 1.0
exclusion 6.879114 0.5669891 -1.1772339 1.0 1.0
exclusion -3.8518734 -6.5558167 -4.5968246 1.5 1.0
exclusion -6.8518734 -3.5558167 2.9031754 1.5 1.0
exclusion 0.6481266 -6.5558167 2.9031754 1.5 1.0
exclusion -8.351873 2.4441833 1.4031754 1.5 1.0
exclusion 5.1481266 5.4441833 -1.5968246 1.5 1.0
exclusion 3.6481266 5.4441833 2.9031754 1.5 1.0
exclusion -0.8518734 6.9441833 -6.0968246 1.5 1.0
exclusion -5.3518734 -0.55581665 5.9031754 1.5 1.0
exclusion 3.6481266 -6.5558167 -3.0968246 1.5 1.0
exclusion 0.6481266 -6.5558167 -6.0968246 1.5 1.0
exclusion 6.6481266 2.4441833 1.4031754 1.5 1.0
exclusion 5.1481266 -5.0558167 1.4031754 1.5 1.0
exclusion 3.6481266 -3.5558167 -7.5968246 1.5 1.0
exclusion -9.851873 -0.55581665 -1.5968246 1.5 1.0
exclusion 5.1481266 0.94418335 -7.5968246 1.5 1.0
exclusion -0.8518734 -2.0558167 7.4031754 1.5 1.0
exclusion 2.1481266 8.444183 -0.096824646 1.5 1.0
exclusion -8.351873 5.4441833 -1.5968246 1.5 1.0
exclusion -2.3518734 2.4441833 7.4031754 1.5 1.0
exclusion -6.8518734 5.4441833 -6.0968246 1.5 1.0
exclusion -3.8518734 -5.0558167 5.9031754 1.5 1.0
exclusion 2.1481266 3.9441833 -9.096825 1.5 1.0
exclusion 2.7676 0.6804 7.2234 1.0 1.0
exclusion 8.148127 0.94418335 -3.0968246 1.5 1.0
exclusion 5.657113 -0.29750106 5.3446264 1.0 1.0
exclusion -2.3518734 0.94418335 -10.596825 1.5 1.0
exclusion -2.3518734 -5.0558167 -9.096825 1.5 1.0
exclusion 6.9096 -2.6437 3.436 1.0 1.0
exclusion -9.851873 2.4441833 -4.5968246 1.5 1.0
exclusion 5.1481266 2.4441833 5.9031754 1.5 1.0
exclusion 0.6481266 -2.0558167 -10.596825 1.5 1.0
exclusion -3.8518734 5.4441833 -9.096825 1.5 1.0
exclusion -6.8518734 2.4441833 -9.096825 1.5 1.0
exclusion -6.8518734 -5.0558167 -7.5968246 1.5 1.0
exclusion -9.851873 -3.5558167 -4.5968246 1.5 1.0
exclusion 3.9393 -3.7184 6.8784 1.0 1.0
exclusion -5.3518734 -2.0558167 -10.596825 1.5 1.0
exclusion -9.851873 -0.55581665 -7.5968246 1.5 1.0
exclusion 8.7841 -0.3911 5.0917 1.0 1.0

AR_agonist_pKi_2.xyz
type x y z tolerance weight additional_flag
H -1.7001194 0.27979022 -0.51461804 1.5 1.0
H 1.0111487 -0.6803593 -3.6627116 1.5 1.0
H 2.1654782 1.5565534 -0.24641228 1.35 1.0 optional
H -1.877025 0.056724504 4.4903493 1.5 1.0 optional
AR -1.2215242 0.060515195 -1.8829569 0.8999999 1.0 optional
HBA 1.3659799 -5.551115E-17 1.110223E-16 1.5 1.0 optional
HBA -0.7545196 -1.0558171 1.9792697 1.5 1.0
HBA -2.517291 0.6468353 -5.0968246 1.5 1.0
HBA -2.517291 0.6468353 -5.0968246 1.5 1.0
HBA -4.2668376 0.2590977 -3.9114542 1.5 1.0 optional
HBD -4.3518734 0.27820322 -3.8643675 1.5 0.8 optional
HBD 2.7931 -0.6282 5.0386 1.0 1.0
exclusion -3.93295 -2.48975 -0.36124998 0.70000005 1.0
exclusion -3.5485935 1.7570832 1.4885219 1.0 1.0
exclusion -0.117598325 2.6081965 2.4300706 0.5500001 1.0
exclusion -5.0400305 3.5107024 -0.20309341 1.0 1.0
exclusion -3.6702645 -2.0525813 2.893721 1.0 1.0
exclusion -0.8518734 5.4441833 -0.096824646 1.5 1.0
exclusion -3.0468028 -2.9509373 -5.4920006 1.0 1.0
exclusion -6.6333 -0.4417 -2.5402 1.0 1.0
exclusion -1.1009195 2.6516218 4.0896034 0.5500001 1.0
exclusion 4.3660417 2.4529862 -2.2251582 1.0 1.0
exclusion 1.3061 2.9907 3.4449 1.0 1.0
exclusion 2.1481266 3.9441833 -4.5968246 1.5 1.0
exclusion -4.4413 2.2664 3.2445 1.0 1.0
exclusion -0.35395026 -0.16883606 -7.2620335 1.0 1.0
exclusion 2.6739 -1.3559 -6.0772 1.0 1.0
exclusion -3.8518734 5.4441833 -3.0968246 1.5 1.0
exclusion 3.8511586 2.0754263 2.1752548 0.70000005 1.0
exclusion -7.2236166 1.6773332 -2.5415668 1.0 1.0
exclusion 5.1481266 -2.0558167 -1.5968246 1.5 1.0
exclusion -0.8518734 -6.5558167 -1.5968246 1.5 1.0
exclusion -2.5424 2.5023 -7.8933 1.0 1.0
exclusion -6.578859 1.615443 -5.644575 1.0 1.0
exclusion -5.3518734 5.4441833 1.4031754 1.5 1.0
exclusion -3.8518734 -6.5558167 1.4031754 1.5 1.0
exclusion -0.8518734 5.4441833 4.4031754 1.5 1.0
exclusion 1.705 -3.7667 4.9132 1.0 1.0
exclusion -6.8518734 -5.0558167 -1.5968246 1.5 1.0
exclusion 6.879114 0.5669891 -1.1772339 1.0 1.0
exclusion -3.8518734 -6.5558167 -4.5968246 1.5 1.0
exclusion -6.8518734 -3.5558167 2.9031754 1.5 1.0
exclusion 0.6481266 -6.5558167 2.9031754 1.5 1.0
exclusion -8.351873 2.4441833 1.4031754 1.5 1.0
exclusion 5.1481266 5.4441833 -1.5968246 1.5 1.0
exclusion 3.6481266 5.4441833 2.9031754 1.5 1.0
exclusion -0.8518734 6.9441833 -6.0968246 1.5 1.0
exclusion -5.3518734 -0.55581665 5.9031754 1.5 1.0
exclusion 3.6481266 -6.5558167 -3.0968246 1.5 1.0
exclusion 0.6481266 -6.5558167 -6.0968246 1.5 1.0
exclusion 6.6481266 2.4441833 1.4031754 1.5 1.0
exclusion 5.1481266 -5.0558167 1.4031754 1.5 1.0
exclusion 3.6481266 -3.5558167 -7.5968246 1.5 1.0
exclusion -9.851873 -0.55581665 -1.5968246 1.5 1.0
exclusion 5.1481266 0.94418335 -7.5968246 1.5 1.0
exclusion -0.8518734 -2.0558167 7.4031754 1.5 1.0
exclusion 2.1481266 8.444183 -0.096824646 1.5 1.0
exclusion -8.351873 5.4441833 -1.5968246 1.5 1.0
exclusion -2.3518734 2.4441833 7.4031754 1.5 1.0
exclusion -6.8518734 5.4441833 -6.0968246 1.5 1.0
exclusion -3.8518734 -5.0558167 5.9031754 1.5 1.0
exclusion 2.1481266 3.9441833 -9.096825 1.5 1.0
exclusion 2.7676 0.6804 7.2234 1.0 1.0
exclusion 8.148127 0.94418335 -3.0968246 1.5 1.0
exclusion 5.657113 -0.29750106 5.3446264 1.0 1.0
exclusion -2.3518734 0.94418335 -10.596825 1.5 1.0
exclusion -2.3518734 -5.0558167 -9.096825 1.5 1.0
exclusion 6.9096 -2.6437 3.436 1.0 1.0
exclusion -9.851873 2.4441833 -4.5968246 1.5 1.0
exclusion 5.1481266 2.4441833 5.9031754 1.5 1.0
exclusion 0.6481266 -2.0558167 -10.596825 1.5 1.0
exclusion -3.8518734 5.4441833 -9.096825 1.5 1.0
exclusion -6.8518734 2.4441833 -9.096825 1.5 1.0
exclusion -6.8518734 -5.0558167 -7.5968246 1.5 1.0
exclusion -9.851873 -3.5558167 -4.5968246 1.5 1.0
exclusion 3.9393 -3.7184 6.8784 1.0 1.0
exclusion -5.3518734 -2.0558167 -10.596825 1.5 1.0
exclusion -9.851873 -0.55581665 -7.5968246 1.5 1.0
exclusion 8.7841 -0.3911 5.0917 1.0 1.0

AR_agonist_pKi_3.xyz
type x y z tolerance weight additional_flag
H -1.7001194 0.27979022 -0.51461804 1.5 1.0

H 1.0111487 -0.6803593 -3.6627116 1.5 1.0
H 2.1654782 1.5565534 -0.24641228 1.35 1.0 optional
H -1.877025 0.056724504 4.4903493 1.5 1.0 optional
AR -1.2215242 0.060515195 -1.8829569 0.8999999 1.0 optional
HBA 1.3659799 -5.551115E-17 1.110223E-16 1.5 1.0 optional
HBA -0.7545196 -1.0558171 1.9792697 1.5 1.0
HBA -2.517291 0.6468353 -5.0968246 1.5 1.0
HBA -2.517291 0.6468353 -5.0968246 1.5 1.0
HBA -4.2668376 0.2590977 -3.9114542 1.5 1.0 optional
HBD -4.3518734 0.27820322 -3.8643675 1.5 0.8 optional
HBD 4.5331 -1.1992 2.9795 1.0 1.0
exclusion -3.93295 -2.48975 -0.36124998 0.70000005 1.0
exclusion -3.5485935 1.7570832 1.4885219 1.0 1.0
exclusion -0.117598325 2.6081965 2.4300706 0.5500001 1.0
exclusion -5.0400305 3.5107024 -0.20309341 1.0 1.0
exclusion -3.6702645 -2.0525813 2.893721 1.0 1.0
exclusion -0.8518734 5.4441833 -0.096824646 1.5 1.0
exclusion -3.0468028 -2.9509373 -5.4920006 1.0 1.0
exclusion -6.6333 -0.4417 -2.5402 1.0 1.0
exclusion -1.1009195 2.6516218 4.0896034 0.5500001 1.0
exclusion 4.3660417 2.4529862 -2.2251582 1.0 1.0
exclusion 1.3061 2.9907 3.4449 1.0 1.0
exclusion 2.1481266 3.9441833 -4.5968246 1.5 1.0
exclusion -4.4413 2.2664 3.2445 1.0 1.0
exclusion -0.35395026 -0.16883606 -7.2620335 1.0 1.0
exclusion 2.6739 -1.3559 -6.0772 1.0 1.0
exclusion -3.8518734 5.4441833 -3.0968246 1.5 1.0
exclusion 3.8511586 2.0754263 2.1752548 0.70000005 1.0
exclusion -7.2236166 1.6773332 -2.5415668 1.0 1.0
exclusion 5.1481266 -2.0558167 -1.5968246 1.5 1.0
exclusion -0.8518734 -6.5558167 -1.5968246 1.5 1.0
exclusion -2.5424 2.5023 -7.8933 1.0 1.0
exclusion -6.578859 1.615443 -5.644575 1.0 1.0
exclusion -5.3518734 5.4441833 1.4031754 1.5 1.0
exclusion -3.8518734 -6.5558167 1.4031754 1.5 1.0
exclusion -0.8518734 5.4441833 4.4031754 1.5 1.0
exclusion 1.705 -3.7667 4.9132 1.0 1.0
exclusion -6.8518734 -5.0558167 -1.5968246 1.5 1.0
exclusion 6.879114 0.5669891 -1.1772339 1.0 1.0
exclusion -3.8518734 -6.5558167 -4.5968246 1.5 1.0
exclusion -6.8518734 -3.5558167 2.9031754 1.5 1.0
exclusion 0.6481266 -6.5558167 2.9031754 1.5 1.0
exclusion -8.351873 2.4441833 1.4031754 1.5 1.0
exclusion 5.1481266 5.4441833 -1.5968246 1.5 1.0
exclusion 3.6481266 5.4441833 2.9031754 1.5 1.0
exclusion -0.8518734 6.9441833 -6.0968246 1.5 1.0
exclusion -5.3518734 -0.55581665 5.9031754 1.5 1.0
exclusion 3.6481266 -6.5558167 -3.0968246 1.5 1.0
exclusion 0.6481266 -6.5558167 -6.0968246 1.5 1.0
exclusion 6.6481266 2.4441833 1.4031754 1.5 1.0
exclusion 5.1481266 -5.0558167 1.4031754 1.5 1.0
exclusion 3.6481266 -3.5558167 -7.5968246 1.5 1.0
exclusion -9.851873 -0.55581665 -1.5968246 1.5 1.0
exclusion 5.1481266 0.94418335 -7.5968246 1.5 1.0
exclusion -0.8518734 -2.0558167 7.4031754 1.5 1.0
exclusion 2.1481266 8.444183 -0.096824646 1.5 1.0
exclusion -8.351873 5.4441833 -1.5968246 1.5 1.0
exclusion -2.3518734 2.4441833 7.4031754 1.5 1.0
exclusion -6.8518734 5.4441833 -6.0968246 1.5 1.0
exclusion -3.8518734 -5.0558167 5.9031754 1.5 1.0
exclusion 2.1481266 3.9441833 -9.096825 1.5 1.0
exclusion 2.7676 0.6804 7.2234 1.0 1.0
exclusion 8.148127 0.94418335 -3.0968246 1.5 1.0
exclusion 5.657113 -0.29750106 5.3446264 1.0 1.0
exclusion -2.3518734 0.94418335 -10.596825 1.5 1.0
exclusion -2.3518734 -5.0558167 -9.096825 1.5 1.0
exclusion 6.9096 -2.6437 3.436 1.0 1.0
exclusion -9.851873 2.4441833 -4.5968246 1.5 1.0
exclusion 5.1481266 2.4441833 5.9031754 1.5 1.0
exclusion 0.6481266 -2.0558167 -10.596825 1.5 1.0
exclusion -3.8518734 5.4441833 -9.096825 1.5 1.0
exclusion -6.8518734 2.4441833 -9.096825 1.5 1.0
exclusion -6.8518734 -5.0558167 -7.5968246 1.5 1.0
exclusion -9.851873 -3.5558167 -4.5968246 1.5 1.0
exclusion 3.9393 -3.7184 6.8784 1.0 1.0
exclusion -5.3518734 -2.0558167 -10.596825 1.5 1.0
exclusion -9.851873 -0.55581665 -7.5968246 1.5 1.0
exclusion 8.7841 -0.3911 5.0917 1.0 1.0

AR_agonist_pKi_4.xyz
type x y z tolerance weight additional_flag
H 3.2584445 -0.5966948 0.18658751 1.5 1.0
H 0.219393 2.086963 -3.724195 1.5 1.0
H 6.2643 2.9099 -1.3666 1.5 1.0
H -1.6543 2.3695 0.878 1.5 1.0 optional
AR 3.3452833 0.36258334 -0.74438334 0.9 1.0
HBA -0.10538763 -0.2157634 -0.6887795 1.15 1.0
HBA 3.0264 2.0808 -4.0087 1.0 1.0
HBA 6.9772263 0.13540444 0.8755529 1.5 1.0
HBA 6.9772263 0.13540444 0.8755529 1.5 1.0
HBD 5.1685 -0.6734 1.0433 1.0 1.0 optional
exclusion 0.6752064 -1.70805 -2.244657 0.3 1.0
exclusion 0.8221855 -2.3757172 1.8267413 0.5500001 1.0
exclusion 3.4293 -2.4079 2.9143 0.70000005 1.0
exclusion 1.1876 4.2405 1.1905 1.0 1.0
exclusion 1.5131068 -0.12550813 3.8889604 1.0 1.0
exclusion 1.279 -2.1889 -4.7712 1.0 1.0
exclusion 5.956312 -3.096695 -2.221096 1.5 1.0
exclusion 0.4609 -4.4358 -0.9249 1.0 1.0
exclusion -2.2735 -2.6673 -1.9683 0.85 1.0
exclusion 6.8406496 0.8041644 -4.534912 1.0 1.0
exclusion 2.9563122 5.903305 -0.72109604 1.5 1.0
exclusion 0.546826 -1.4462416 -6.034606 0.70000005 1.0
exclusion 7.4595 -2.6503 0.8169 0.70000005 1.0
exclusion 7.5084 -0.6356 -3.8889 0.70000005 1.0
exclusion 7.4179 -2.0806 2.1303 0.70000005 1.0
exclusion 2.9563122 4.403305 3.778904 1.5 1.0
exclusion 2.9563122 -6.096695 0.77890396 1.5 1.0
exclusion 6.5004 -3.8586 2.3992 1.0 1.0
exclusion -0.42999417 -5.4383593 -2.572224 1.0 1.0
exclusion -3.8693948 -1.5261428 0.6058817 0.70000005 1.0
exclusion 9.2478 -0.812 -0.8059 1.0 1.0
exclusion -0.5141 -3.4554 -5.6902 0.5500001 1.0
exclusion 7.4945655 -0.99537516 3.8891528 1.0 1.0
exclusion 2.9563122 -6.096695 -3.721096 1.5 1.0
exclusion -1.5436878 -4.596695 2.278904 1.5 1.0
exclusion 4.456312 -3.096695 -6.721096 1.5 1.0
exclusion -2.2919235 1.2855067 -5.817031 0.5500001 1.0
exclusion -1.5436878 -0.09669495 5.278904 1.5 1.0
exclusion 5.956312 5.903305 -3.721096 1.5 1.0
exclusion -3.7634 4.1692 -0.8317 1.0 1.0
exclusion -4.350715 -0.082826495 2.2720928 0.70000005 1.0
exclusion 2.9563122 -0.09669495 6.778904 1.5 1.0
exclusion 9.2695 2.72 1.6221 1.0 1.0
exclusion 9.9252 1.9595 -0.1488 1.0 1.0
exclusion 1.4563122 -4.596695 5.278904 1.5 1.0
exclusion 4.456312 1.403305 -8.221096 1.5 1.0
exclusion -0.04368782 -1.596695 -8.221096 1.5 1.0
exclusion -5.5488 -1.0231 -0.8747 1.0 1.0
exclusion -4.3876 -2.6105 -4.2275 0.5500001 1.0
exclusion -0.04368782 7.403305 -3.721096 1.5 1.0
exclusion -0.04368782 7.403305 2.278904 1.5 1.0
exclusion 2.9563122 5.903305 -6.721096 1.5 1.0
exclusion -3.0436878 4.403305 -5.221096 1.5 1.0
exclusion -0.04368782 2.903305 -8.221096 1.5 1.0
exclusion -1.5436878 4.403305 5.278904 1.5 1.0
exclusion 5.956312 -6.096695 3.778904 1.5 1.0
exclusion 8.956312 -3.096695 -5.221096 1.5 1.0
exclusion -4.543688 -4.596695 -0.72109604 1.5 1.0
exclusion -4.543688 4.403305 2.278904 1.5 1.0
exclusion 8.956312 1.403305 -6.721096 1.5 1.0
exclusion 7.456312 4.403305 5.278904 1.5 1.0

exclusion 7.456312 7.403305 0.77890396 1.5 1.0
exclusion 8.956312 -6.096695 -0.72109604 1.5 1.0
exclusion -6.043688 -0.09669495 -3.721096 1.5 1.0
exclusion -3.0436878 7.403305 -0.72109604 1.5 1.0
exclusion 10.456312 4.403305 -3.721096 1.5 1.0
exclusion -6.043688 4.403305 -2.221096 1.5 1.0
exclusion -6.043688 -0.09669495 3.778904 1.5 1.0
exclusion 11.956312 -3.096695 -2.221096 1.5 1.0
exclusion 11.956312 -3.096695 2.278904 1.5 1.0
exclusion 10.456312 1.403305 6.778904 1.5 1.0
AR_agonist_pKi_5.xyz
type x y z tolerance weight additional_flag
H -1.34128 0.27870655 0.17449707 1.65 0.79999995
H -0.92364997 -1.12815 3.2632499 1.475 0.79999995
H -4.5511 -1.7566 1.1545 1.5 1.0
HBA -0.019875329 3.286779 2.2467232 1.5 0.9
HBA -1.3355 -1.0864 3.7493 1.15 0.79999995 optional
HBA 2.2645416 0.55866444 6.335511 1.5 1.0 optional
HBA -3.4634752 -0.85143393 -3.0877333 1.5 1.0
HBD 2.7018173 0.16188556 6.386695 1.0250001 0.8
exclusion -1.4634752 5.6485662 -2.5877333 1.5 1.0
exclusion -5.963475 4.1485662 4.9122667 1.5 1.0
exclusion -7.463475 -0.35143375 3.4122667 1.5 1.0
exclusion -7.463475 2.6485662 0.41226673 1.5 1.0
exclusion -4.463475 -0.35143375 7.9122667 1.5 1.0
exclusion -4.463475 7.1485662 1.9122667 1.5 1.0
exclusion -2.9634752 4.1485662 7.9122667 1.5 1.0
exclusion 6.036525 -1.8514338 1.9122667 1.5 1.0
exclusion 3.0365248 -4.8514338 -1.0877333 1.5 1.0
exclusion 3.0365248 5.6485662 -2.5877333 1.5 1.0
exclusion 6.036525 2.6485662 -1.0877333 1.5 1.0
exclusion 6.036525 4.1485662 3.4122667 1.5 1.0
exclusion -2.9634752 2.6485662 -5.5877333 1.5 1.0
exclusion 0.036524773 8.648566 0.41226673 1.5 1.0
exclusion -5.963475 5.6485662 -2.5877333 1.5 1.0
exclusion -1.4634752 8.648566 4.9122667 1.5 1.0
exclusion -1.4634752 -3.3514338 9.412267 1.5 1.0
exclusion 3.0365248 8.648566 3.4122667 1.5 1.0
exclusion 1.5365248 7.1485662 7.9122667 1.5 1.0
exclusion -1.4634752 1.1485662 10.912267 1.5 1.0
exclusion 3.1463 -1.3839 9.7072 1.15 1.0
exclusion -8.963475 -3.3514338 0.41226673 1.5 1.0
exclusion 3.0365248 -4.8514338 7.9122667 1.5 1.0
exclusion -8.963475 -0.35143375 -2.5877333 1.5 1.0
exclusion 6.036525 -4.8514338 4.9122667 1.5 1.0
exclusion 6.036525 -1.8514338 7.9122667 1.5 1.0
exclusion 6.036525 4.1485662 7.9122667 1.5 1.0
exclusion 1.5365248 4.1485662 10.912267 1.5 1.0
exclusion 1.5365248 -3.3514338 -7.0877333 1.5 1.0
exclusion -1.4634752 -6.3514338 -5.5877333 1.5 1.0
exclusion 0.036524773 1.1485662 -8.587733 1.5 1.0
exclusion 9.036525 -0.35143375 4.9122667 1.5 1.0
exclusion 4.536525 1.1485662 10.912267 1.5 1.0
exclusion -2.9634752 -1.8514338 -8.587733 1.5 1.0
exclusion -8.963475 2.6485662 -5.5877333 1.5 1.0
exclusion 1.5365248 -1.8514338 12.412267 1.5 1.0
exclusion -8.963475 -4.8514338 -4.0877333 1.5 1.0
exclusion -5.963475 1.1485662 -8.587733 1.5 1.0
exclusion -5.963475 -4.8514338 -7.0877333 1.5 1.0
exclusion -8.963475 -1.8514338 -7.0877333 1.5 1.0
AR_agonist_pKi_6.xyz
type x y z tolerance weight additional_flag
H 3.2584445 -0.5966948 0.18658751 1.5 1.0
H 5.538154 1.4239805 -1.9566746 1.5 0.8888889
H 1.0291 2.1836 -3.4243 1.5 1.0
H -1.6543 2.3695 0.878 1.5 1.0 optional
AR 3.2405105 -0.18585388 -0.3379436 0.8999999 1.0 optional
AR 3.6529207 1.414057 -2.1158662 0.8999999 0.5555556
optional
HBA -1.29125 0.081 -0.8679 1.3 1.0
HBA 6.9772263 0.13540444 0.8755529 1.5 1.0
HBA 6.9772263 0.13540444 0.8755529 1.5 1.0
HBD 5.1685 -0.6734 1.0433 1.0 1.0 optional
exclusion 0.6752064 -1.70805 -2.244657 0.3 1.0
exclusion 0.8221855 -2.3757172 1.8267413 0.5500001 1.0
exclusion 3.4293 -2.4079 2.9143 0.70000005 1.0
exclusion 1.1876 4.2405 1.1905 1.0 1.0
exclusion 1.5131068 -0.12550813 3.8889604 1.0 1.0
exclusion 1.279 -2.1889 -4.7712 1.0 1.0
exclusion 5.956312 -3.096695 -2.221096 1.5 1.0
exclusion 0.4609 -4.4358 -0.9249 1.0 1.0
exclusion -2.2735 -2.6673 -1.9683 1.0 1.0
exclusion 6.8406496 0.8041644 -4.534912 1.0 1.0
exclusion 2.9563122 5.903305 -0.72109604 1.5 1.0
exclusion 0.546826 -1.4462416 -6.034606 0.70000005 1.0
exclusion 7.4595 -2.6503 0.8169 0.70000005 1.0
exclusion 7.5084 -0.6356 -3.8889 0.70000005 1.0
exclusion 7.4179 -2.0806 2.1303 0.70000005 1.0
exclusion 2.9563122 4.403305 3.778904 1.5 1.0
exclusion 2.9563122 -6.096695 0.77890396 1.5 1.0
exclusion 6.5004 -3.8586 2.3992 1.0 1.0
exclusion -0.42999417 -5.4383593 -2.572224 1.0 1.0
exclusion -3.8693948 -1.5261428 0.6058817 0.70000005 1.0
exclusion 9.2478 -0.812 -0.8059 1.0 1.0
exclusion -0.5141 -3.4554 -5.6902 0.5500001 1.0
exclusion 7.4945655 -0.99537516 3.8891528 1.0 1.0
exclusion 2.9563122 -6.096695 -3.721096 1.5 1.0
exclusion -1.5436878 -4.596695 2.278904 1.5 1.0
exclusion 4.456312 -3.096695 -6.721096 1.5 1.0
exclusion -2.2919235 1.2855067 -5.817031 0.5500001 1.0
exclusion -1.5436878 -0.09669495 5.278904 1.5 1.0
exclusion 5.956312 5.903305 -3.721096 1.5 1.0
exclusion -3.7634 4.1692 -0.8317 1.0 1.0
exclusion -4.350715 -0.082826495 2.2720928 0.70000005 1.0
exclusion 2.9563122 -0.09669495 6.778904 1.5 1.0
exclusion 9.2695 2.72 1.6221 1.0 1.0
exclusion 9.9252 1.9595 -0.1488 1.0 1.0
exclusion 1.4563122 -4.596695 5.278904 1.5 1.0
exclusion 4.456312 1.403305 -8.221096 1.5 1.0
exclusion -0.04368782 -1.596695 -8.221096 1.5 1.0
exclusion -5.5488 -1.0231 -0.8747 1.0 1.0
exclusion -4.3876 -2.6105 -4.2275 0.5500001 1.0
exclusion -0.04368782 7.403305 -3.721096 1.5 1.0
exclusion -0.04368782 7.403305 2.278904 1.5 1.0
exclusion -3.0436878 4.403305 -5.221096 1.5 1.0
exclusion 2.9563122 5.903305 -6.721096 1.5 1.0
exclusion -0.04368782 2.903305 -8.221096 1.5 1.0
exclusion 5.956312 -6.096695 3.778904 1.5 1.0
exclusion -1.5436878 4.403305 5.278904 1.5 1.0
exclusion 8.956312 -3.096695 -5.221096 1.5 1.0
exclusion -4.543688 -4.596695 -0.72109604 1.5 1.0
exclusion -4.543688 4.403305 2.278904 1.5 1.0
exclusion 8.956312 1.403305 -6.721096 1.5 1.0
exclusion 7.456312 4.403305 5.278904 1.5 1.0
exclusion 7.456312 7.403305 0.77890396 1.5 1.0
exclusion 8.956312 -6.096695 -0.72109604 1.5 1.0
exclusion -6.043688 -0.09669495 -3.721096 1.5 1.0
exclusion -3.0436878 7.403305 -0.72109604 1.5 1.0
exclusion 10.456312 4.403305 -3.721096 1.5 1.0
exclusion -6.043688 4.403305 -2.221096 1.5 1.0
exclusion -6.043688 -0.09669495 3.778904 1.5 1.0
exclusion 11.956312 -3.096695 -2.221096 1.5 1.0
exclusion 11.956312 -3.096695 2.278904 1.5 1.0
exclusion 10.456312 1.403305 6.778904 1.5 1.0
AR_agonist_pKi_7.xyz
type x y z tolerance weight additional_flag
H 3.2584445 -0.5966948 0.18658751 1.5 1.0
H 0.49229652 2.4328814 -3.0908976 1.5 1.0
H 6.2643 2.9099 -1.3666 1.5 1.0
H -1.6543 2.3695 0.878 1.5 1.0 optional
AR 3.3452833 0.36258334 -0.74438334 0.9 1.0
AR 5.6331334 0.5085833 0.14336668 0.9 1.0

HBA 0.13455619 -0.5106817 -0.40198973 1.075 1.0
HBA 6.9772263 0.13540444 0.8755529 1.5 1.0
HBA 6.9772263 0.13540444 0.8755529 1.5 1.0
HBD 5.1685 -0.6734 1.0433 1.0 1.0 optional
exclusion 0.6752064 -1.70805 -2.244657 0.3 1.0
exclusion 0.8221855 -2.3757172 1.8267413 0.5500001 1.0
exclusion 3.4293 -2.4079 2.9143 0.70000005 1.0
exclusion 1.5131068 -0.12550813 3.8889604 1.0 1.0
exclusion 1.1876 4.2405 1.1905 1.0 1.0
exclusion 5.956312 -3.096695 -2.221096 1.5 1.0
exclusion 2.5624974 4.9280396 -1.4910501 0.75 1.0
exclusion 1.279 -2.1889 -4.7712 1.0 1.0
exclusion 0.4609 -4.4358 -0.9249 1.0 1.0
exclusion 5.4564 2.2894 -5.137 0.70000005 1.0
exclusion 6.8406496 0.8041644 -4.534912 1.0 1.0
exclusion 7.4595 -2.6503 0.8169 0.70000005 1.0
exclusion -2.2735 -2.6673 -1.9683 0.85 1.0
exclusion 7.5084 -0.6356 -3.8889 0.70000005 1.0
exclusion 7.4179 -2.0806 2.1303 0.70000005 1.0
exclusion 0.546826 -1.4462416 -6.034606 0.70000005 1.0
exclusion 2.9563122 4.403305 3.778904 1.5 1.0
exclusion 6.5004 -3.8586 2.3992 1.0 1.0
exclusion 2.9563122 -6.096695 0.77890396 1.5 1.0
exclusion 9.2478 -0.812 -0.8059 1.0 1.0
exclusion -0.42999417 -5.4383593 -2.572224 1.0 1.0
exclusion 7.4945655 -0.99537516 3.8891528 1.0 1.0
exclusion -3.8693948 -1.5261428 0.6058817 0.70000005 1.0
exclusion -0.5141 -3.4554 -5.6902 0.5500001 1.0
exclusion 2.9563122 -6.096695 -3.721096 1.5 1.0
exclusion -1.5436878 -4.596695 2.278904 1.5 1.0
exclusion 4.456312 -3.096695 -6.721096 1.5 1.0
exclusion 5.956312 5.903305 -3.721096 1.5 1.0
exclusion -1.5436878 -0.09669495 5.278904 1.5 1.0
exclusion 9.2695 2.72 1.6221 1.0 1.0
exclusion 2.9563122 -0.09669495 6.778904 1.5 1.0
exclusion 9.9252 1.9595 -0.1488 1.0 1.0
exclusion -3.7634 4.1692 -0.8317 1.0 1.0
exclusion -4.350715 -0.082826495 2.2720928 0.70000005 1.0
exclusion 1.4563122 -4.596695 5.278904 1.5 1.0
exclusion 4.456312 1.403305 -8.221096 1.5 1.0
exclusion -0.04368782 -1.596695 -8.221096 1.5 1.0
exclusion -5.5488 -1.0231 -0.8747 1.0 1.0
exclusion -0.04368782 7.403305 2.278904 1.5 1.0
exclusion -0.04368782 7.403305 -3.721096 1.5 1.0
exclusion 2.9563122 5.903305 -6.721096 1.5 1.0
exclusion 5.956312 -6.096695 3.778904 1.5 1.0
exclusion -4.3876 -2.6105 -4.2275 0.5500001 1.0
exclusion 8.956312 -3.096695 -5.221096 1.5 1.0
exclusion -3.0436878 4.403305 -5.221096 1.5 1.0
exclusion -0.04368782 2.903305 -8.221096 1.5 1.0
exclusion -1.5436878 4.403305 5.278904 1.5 1.0
exclusion -4.543688 -4.596695 -0.72109604 1.5 1.0
exclusion 7.456312 4.403305 5.278904 1.5 1.0
exclusion 8.956312 1.403305 -6.721096 1.5 1.0
exclusion 7.456312 7.403305 0.77890396 1.5 1.0
exclusion 8.956312 -6.096695 -0.72109604 1.5 1.0
exclusion -4.543688 4.403305 2.278904 1.5 1.0
exclusion -6.043688 -0.09669495 -3.721096 1.5 1.0
exclusion -3.0436878 7.403305 -0.72109604 1.5 1.0
exclusion 10.456312 4.403305 -3.721096 1.5 1.0
exclusion -6.043688 -0.09669495 3.778904 1.5 1.0
exclusion -6.043688 4.403305 -2.221096 1.5 1.0
exclusion 11.956312 -3.096695 -2.221096 1.5 1.0
exclusion 11.956312 -3.096695 2.278904 1.5 1.0
exclusion 10.456312 1.403305 6.778904 1.5 1.0

AR_agonist_pKi_8.xyz
type x y z tolerance weight additional_flag
H 0.8132 2.6514 -2.9695 1.65 1.0 optional
H -1.2100599 -0.96596044 -3.6484063 1.2 1.0 optional
H 6.2643 2.9099 -1.3666 1.5 1.0
H -2.6573 0.5737 -1.1608 0.9000001 1.0 optional
AR 3.1588833 0.4086 -0.89596665 0.9 1.0
AR 5.6331334 0.5085833 0.14336668 0.9 1.0
HBA 6.9772263 0.13540444 0.8755529 1.5 1.0
HBA 6.9772263 0.13540444 0.8755529 1.5 1.0
HBD 0.3618781 -0.60609084 -0.26339486 1.0375 1.0
HBD 5.1685 -0.6734 1.0433 1.0 1.0 optional
exclusion 2.7049987 -2.8022203 0.3382277 0.5500001 1.0
exclusion 1.5776782 -2.0284271 1.3728259 0.40000007 1.0
exclusion 0.26070845 -3.1098564 -0.31139922 0.79999995 1.0
exclusion 1.411428 -1.6956403 -4.138379 0.3 1.0
exclusion 0.3116014 -2.7836537 -2.4785357 0.3 1.0
exclusion -0.866022 -2.2690651 -1.4176152 0.3 1.0
exclusion -0.83237815 1.1296759 1.2017852 0.40000007 1.0
exclusion 5.095967 -2.5869017 0.9038878 0.5500001 1.0
exclusion 1.5779581 3.9840655 -0.16736549 0.40000007 1.0
exclusion -1.4812236 -0.38520342 0.81282264 0.3 1.0
exclusion -1.6794137 -1.1599922 -0.034049213 0.3 1.0
exclusion 2.141247 4.300698 -1.762455 0.33750004 1.0
exclusion 3.4293 -2.4079 2.9143 0.40000007 1.0
exclusion -2.068098 -1.2382709 -0.6885412 0.3 1.0
exclusion 1.5131068 -0.12550813 3.8889604 1.0 1.0
exclusion 5.956312 -3.096695 -2.221096 1.5 1.0
exclusion 0.912913 -1.8175708 -5.402903 0.3 1.0
exclusion 5.4564 2.2894 -5.137 0.70000005 1.0
exclusion 6.8406496 0.8041644 -4.534912 1.0 1.0
exclusion -2.1504378 -2.5071254 -3.7297456 0.3 1.0
exclusion 7.4595 -2.6503 0.8169 0.70000005 1.0
exclusion 1.2861247 2.5475392 -6.150199 0.475 1.0
exclusion 7.5084 -0.6356 -3.8889 0.70000005 1.0
exclusion 7.4179 -2.0806 2.1303 0.70000005 1.0
exclusion 0.3703595 2.9288347 -6.056521 0.40000007 1.0
exclusion 2.9563122 -6.096695 0.77890396 1.5 1.0
exclusion -3.9054086 -0.69276834 -0.9777765 0.3 1.0
exclusion -0.42999417 -5.4383593 -2.572224 1.0 1.0
exclusion 2.9563122 4.403305 3.778904 1.5 1.0
exclusion 6.5004 -3.8586 2.3992 0.85 1.0
exclusion -0.5141 -3.4554 -5.6902 0.5500001 1.0
exclusion -3.8693948 -1.5261428 0.6058817 0.40000007 1.0
exclusion 2.9563122 -6.096695 -3.721096 1.5 1.0
exclusion -1.5436878 -4.596695 2.278904 1.5 1.0
exclusion 9.2478 -0.812 -0.8059 1.0 1.0
exclusion 4.456312 -3.096695 -6.721096 1.5 1.0
exclusion 7.4945655 -0.99537516 3.8891528 1.0 1.0
exclusion -1.5436878 -0.09669495 5.278904 1.5 1.0
exclusion -4.350715 -0.082826495 2.2720928 0.70000005 1.0
exclusion 5.956312 5.903305 -3.721096 1.5 1.0
exclusion 2.9563122 -0.09669495 6.778904 1.5 1.0
exclusion 1.4563122 -4.596695 5.278904 1.5 1.0
exclusion 4.456312 1.403305 -8.221096 1.5 1.0
exclusion 9.2695 2.72 1.6221 1.0 1.0
exclusion 9.9252 1.9595 -0.1488 1.0 1.0
exclusion -0.04368782 -1.596695 -8.221096 1.5 1.0
exclusion -4.3876 -2.6105 -4.2275 0.85 1.0
exclusion -5.5488 -1.0231 -0.8747 1.0 1.0
exclusion -3.0436878 4.403305 -5.221096 0.60000014 1.0
exclusion -0.04368782 2.903305 -8.221096 1.5 1.0
exclusion -4.543688 -4.596695 -0.72109604 1.5 1.0
exclusion -0.04368782 7.403305 -3.721096 1.0500001 1.0
exclusion 2.9563122 5.903305 -6.721096 1.5 1.0
exclusion -0.04368782 7.403305 2.278904 1.5 1.0
exclusion 5.956312 -6.096695 3.778904 1.5 1.0
exclusion 8.956312 -3.096695 -5.221096 1.5 1.0
exclusion -1.5436878 4.403305 5.278904 1.5 1.0
exclusion -4.543688 4.403305 2.278904 1.5 1.0
exclusion -6.043688 -0.09669495 -3.721096 1.5 1.0
exclusion 8.956312 1.403305 -6.721096 1.5 1.0
exclusion 8.956312 -6.096695 -0.72109604 1.5 1.0
exclusion 7.456312 4.403305 5.278904 1.5 1.0
exclusion 7.456312 7.403305 0.77890396 1.5 1.0
exclusion -3.0436878 7.403305 -0.72109604 1.5 1.0

exclusion -6.043688 4.403305 -2.221096 1.5 1.0
exclusion -6.043688 -0.09669495 3.778904 1.5 1.0
exclusion 10.456312 4.403305 -3.721096 1.5 1.0
exclusion 11.956312 -3.096695 -2.221096 1.5 1.0
exclusion 11.956312 -3.096695 2.278904 1.5 1.0
exclusion 10.456312 1.403305 6.778904 1.5 1.0

AR_agonist_pKi_9.xyz
type x y z tolerance weight additional_flag
H 0.7821676 0.54710287 -3.9959369 1.5 1.0
H -3.0296412 -0.47392425 -6.5054703 1.5 1.0
H -1.6239414 0.74701 0.26291734 1.5 1.0
AR 0.33102262 0.51067054 -4.1728907 0.8999999 1.0
AR 0.04975804 0.35514832 -6.612386 0.8999999 1.0
AR -1.3876553 0.67787683 -0.21738094 0.8999999 0.9411765
HBA 1.177459 0.5631988 -9.026758 1.5 1.0
HBA 1.4155989 0.5353879 -6.7443023 1.5 1.0
exclusion -4.240395 -1.8740256 -3.7364535 1.0 1.0
exclusion -3.309 -2.0721 -1.2725 1.0 1.0
exclusion -1.0296412 4.3582516 -5.526758 1.5 1.0
exclusion -1.0296412 -6.1417484 -4.026758 1.5 1.0
exclusion 3.4703588 -3.1417484 -7.026758 1.5 1.0
exclusion 4.970359 -1.6417484 -2.5267582 1.5 1.0
exclusion -4.029641 5.8582516 -2.5267582 1.5 1.0
exclusion -2.5296412 -6.1417484 -8.526758 1.5 1.0
exclusion -5.529641 -6.1417484 -4.026758 1.5 1.0
exclusion -8.529641 -1.6417484 -4.026758 1.5 1.0
exclusion -5.529641 2.8582516 -10.026758 1.5 1.0
exclusion 4.970359 4.3582516 -5.526758 1.5 1.0
exclusion 1.9703588 5.8582516 -8.526758 1.5 1.0
exclusion -8.529641 1.3582516 -7.026758 1.5 1.0
exclusion 3.4703588 -6.1417484 -2.5267582 1.5 1.0
exclusion 1.9703588 7.3582516 -4.026758 1.5 1.0
exclusion -5.529641 -4.6417484 0.4732418 1.5 1.0
exclusion 0.47035885 -6.1417484 0.4732418 1.5 1.0
exclusion 6.470359 -0.14174843 -7.026758 1.5 1.0
exclusion -8.529641 2.8582516 -2.5267582 1.5 1.0
exclusion -2.5296412 5.8582516 -10.026758 1.5 1.0
exclusion -7.029641 5.8582516 -5.526758 1.5 1.0
exclusion 4.970359 2.8582516 -10.026758 1.5 1.0
exclusion 1.9703588 -6.1417484 -10.026758 1.5 1.0
exclusion -8.529641 -0.14174843 0.4732418 1.5 1.0
exclusion -1.0296412 7.3582516 0.4732418 1.5 1.0
exclusion -8.529641 -4.6417484 -7.026758 1.5 1.0
exclusion -2.5296412 2.8582516 -13.026758 1.5 1.0
exclusion -5.529641 -1.6417484 3.4732418 1.5 1.0
exclusion -2.5296412 -4.6417484 3.4732418 1.5 1.0
exclusion -8.529641 -1.6417484 -10.026758 1.5 1.0
exclusion -5.529641 -4.6417484 -11.526758 1.5 1.0
exclusion -5.529641 -0.14174843 -13.026758 1.5 1.0
exclusion -7.029641 5.8582516 0.4732418 1.5 1.0
exclusion -1.0296412 -4.6417484 -13.026758 1.5 1.0
exclusion 6.470359 -3.1417484 -10.026758 1.5 1.0
exclusion 6.470359 -0.14174843 1.9732418 1.5 1.0
exclusion 0.47035885 2.8582516 4.973242 1.5 1.0
exclusion 4.970359 -4.6417484 1.9732418 1.5 1.0
exclusion 0.47035885 -0.14174843 -14.526758 1.5 1.0
exclusion 3.4703588 -3.1417484 -13.026758 1.5 1.0
exclusion -7.029641 2.8582516 3.4732418 1.5 1.0
exclusion -4.029641 5.8582516 3.4732418 1.5 1.0
exclusion 0.47035885 5.8582516 -13.026758 1.5 1.0
exclusion 3.4703588 -0.14174843 4.973242 1.5 1.0
exclusion 1.9703588 -4.6417484 4.973242 1.5 1.0
exclusion -1.0296412 -1.6417484 6.473242 1.5 1.0
exclusion -4.029641 1.3582516 6.473242 1.5 1.0
exclusion 3.4703588 2.8582516 -14.526758 1.5 1.0
exclusion 6.470359 -0.14174843 -13.026758 1.5 1.0
